# Parity induces differentiation and reduces Wnt/Notch signaling ratio and proliferation potential of basal stem/progenitor cells isolated from mouse mammary epithelium

**DOI:** 10.1186/bcr3419

**Published:** 2013-04-29

**Authors:** Fabienne Meier-Abt, Emanuela Milani, Tim Roloff, Heike Brinkhaus, Stephan Duss, Dominique S Meyer, Ina Klebba, Piotr J Balwierz, Erik van Nimwegen, Mohamed Bentires-Alj

**Affiliations:** 1 Mechanisms of Cancer, Friedrich Miescher Institute for Biomedical Research (FMI), Maulbeerstrasse 66, Basel, CH-4058, Switzerland; 2 Philosophical Natural Sciences, University of Basel, Klingelbergstrasse 50, Basel, CH-4056, Switzerland; 3 Functional Genomics, Friedrich Miescher Institute for Biomedical Research (FMI), Maulbeerstrasse 66, Basel, CH-4058, Switzerland; 4 Biozentrum, University of Basel, Klingelbergstrasse 70, Basel, CH-4056, Switzerland

## Abstract

**Introduction:**

Early pregnancy has a strong protective effect against breast cancer in humans and rodents, but the underlying mechanism is unknown. Because breast cancers are thought to arise from specific cell subpopulations of mammary epithelia, we studied the effect of parity on the transcriptome and the differentiation/proliferation potential of specific luminal and basal mammary cells in mice.

**Methods:**

Mammary epithelial cell subpopulations (luminal Sca1^-^, luminal Sca1^+^, basal stem/progenitor, and basal myoepithelial cells) were isolated by flow cytometry from parous and age-matched virgin mice and examined by using a combination of unbiased genomics, bioinformatics, *in vitro *colony formation, and *in vivo *limiting dilution transplantation assays. Specific findings were further investigated with immunohistochemistry in entire glands of parous and age-matched virgin mice.

**Results:**

Transcriptome analysis revealed an upregulation of differentiation genes and a marked decrease in the Wnt/Notch signaling ratio in basal stem/progenitor cells of parous mice. Separate bioinformatics analyses showed reduced activity for the canonical Wnt transcription factor LEF1/TCF7 and increased activity for the Wnt repressor TCF3. This finding was specific for basal stem/progenitor cells and was associated with downregulation of potentially carcinogenic pathways and a reduction in the proliferation potential of this cell subpopulation *in vitro *and *in vivo*. As a possible mechanism for decreased Wnt signaling in basal stem/progenitor cells, we found a more than threefold reduction in the expression of the secreted Wnt ligand *Wnt4 *in total mammary cells from parous mice, which corresponded to a similar decrease in the proportion of Wnt4-secreting and estrogen/progesterone receptor-positive cells. Because recombinant Wnt4 rescued the proliferation defect of basal stem/progenitor cells *in vitro*, reduced Wnt4 secretion appears to be causally related to parity-induced alterations of basal stem/progenitor cell properties in mice.

**Conclusions:**

By revealing that parity induces differentiation and downregulates the Wnt/Notch signaling ratio and the *in vitro *and *in vivo *proliferation potential of basal stem/progenitor cells in mice, our study sheds light on the long-term consequences of an early pregnancy. Furthermore, it opens the door to future studies assessing whether inhibitors of the Wnt pathway may be used to mimic the parity-induced protective effect against breast cancer.

## Introduction

Pregnancy is the most significant modifiable factor known for breast cancer risk in women. Although an initial increase in risk occurs immediately after parturition in women older than 25 years, the overall lifetime risk of breast cancer decreases after pregnancy [1,2]. This protective effect is > 50% if a full-term pregnancy has occurred before the age of 20 years [1]. Similarly, pregnancy and pregnancy-mimicking hormones have a strong protective effect against mammary tumors in rodents. This is true both for carcinogen-induced mammary tumors [3] and for genetically engineered mouse models of breast cancer [4].

The cellular and molecular mechanisms underlying the breast cancer-protective effect of early pregnancy remain unclear. Frequently raised hypotheses involve cell nonautonomous mechanisms such as systemic changes in circulating hormones and/or changes in the stromal composition of the mammary gland [5,6], and cell autonomous processes such as changes in the differentiation state of mammary epithelial cells [7]. Furthermore, numerous parity-induced changes in gene expression have been identified in genome-wide expression profiles of entire lobular breast tissues of women or entire mammary glands of rats and mice [8-10]. However, it is not known to what extent these tissue studies reflect alterations in gene-expression profiles of distinct mammary epithelial cell subpopulations. Hence, given that breast cancers arise from specific subpopulations of mammary epithelial cells [11], investigations of early parity-induced gene-expression changes in distinct mammary epithelial cell subpopulations are warranted.

The mammary epithelium is hierarchically organized into differentiated luminal and basal (myoepithelial) cells, luminal and basal progenitor cells, and mammary stem cells [12,13]. Whereas the latter were originally thought to lie exclusively in the basal compartment and to be multipotent (able to form both luminal and basal epithelial cells), recent lineage-tracing experiments indicated the existence of unipotent basal and luminal mammary stem cells and identified multipotent mammary stem cells solely in the embryonic and possibly in the pregnant gland [14,15]. Distinct mammary epithelial cell subpopulations, including luminal progenitor and basal stem/progenitor cells can be isolated with fluorescence-activated cell sorting (FACS) by using specific cell-surface markers from both parous and virgin mice [16-21]. Whereas progenitor cells in general can be characterized *in vitro *by their colony-forming potential [16,22,23], the basal stem/progenitor cell subpopulation has the additional capacity to repopulate deepithelialized mouse mammary fat pads *in vivo *[16-18]. Although previous studies in total mammary epithelial cells indicated either no change or a decrease in the mammary repopulating capacity after parity [20,21], the consequences of parity on the transcriptome and functionality of specific mammary epithelial cell subpopulations have not been investigated.

Therefore, we examined in this study whether pregnancy alters the gene-expression profiles ("gene signature") and the differentiation/proliferation potentials of the various mammary epithelial cell subpopulations. The results indicate that early parity decreases *Wnt4 *expression in luminal epithelial cells, leading to a reduction in the Wnt/Notch signaling ratio specifically in basal stem/progenitor cells. As expected, the decrease in the Wnt/Notch signaling ratio is associated with a concomitant strong prodifferentiation and antiproliferation phenotype in basal stem/progenitor cells. Because a decrease in Wnt signaling is known to have an anticarcinogenic effect [24,25], the findings support the hypothesis that a reduction in the Wnt/Notch signaling ratio in basal mammary stem/progenitor cells plays a role in the mitigating effect of early pregnancy on breast tumorigenesis.

## Methods

### Animals and animal experimentation

All experiments were conducted in genetically homogenous FVB/NHanHsd mice purchased from Harlan Laboratories. The mice were bred and maintained in the animal facility of the Friedrich Miescher Institute, according to the Swiss guidelines on animal experimentation. All experiments were performed under permit 2159-2, in accordance with the animal-welfare ordinance and approved by the cantonal veterinary office of Basel Stadt, Switzerland. For the early-pregnancy protocol, mice were time-mated when 42 days old and allowed to lactate for 21 days. The postweaning period until cell harvest was 40 days, unless stated otherwise. To control for the estrus cycle in the transcriptome analyses, at least five mice were grouped for gland harvesting. For immunohistochemical analyses and determination of blood progesterone levels, mice in estrus were used, as assessed by the presence of a vaginal plug after an overnight mating. Age-matched virgin control mice were maintained under the same conditions as parous mice.

### Whole mounts

Whole mounts were prepared by fixing the glands on glass slides with methacarnoy solution (60% methanol, 30% chloroform, 10% glacial acetic acid) for 4 hours at room temperature. The mounts were hydrated by sequential incubation in ethanol solutions of decreasing concentration: 100%, (overnight), 70%, 50%, and 30% (15 minutes each), distilled water (2 × 5 minutes), and stained overnight with an aqueous solution of 2% carmine (Sigma, Buchs, Switzerland) and 5% aluminum potassium sulfate (Sigma, Buchs, Switzerland). The mounts were dehydrated in ethanol solutions (70%, 90%, 95%, and 2 × 100%, for 15 minutes each) and cleared with xylene overnight. Images were captured with an Epson Expression 1600 Pro scanner.

### Mammary cell preparation

The fourth mammary glands from virgin and parous mice were collected after lymph node removal and pooled. Mammary epithelial organoids were prepared as described [26]. Adipocytes were removed by repeated centrifugations (300 *g*). Red blood cells (RBCs) were eliminated by incubation with 8.3 g/L ammonium chloride (pH 7.5) for 5 minutes. The number of fibroblasts in gland extracts was reduced by their attachment to polystyrene cell-culture flasks (Corning, Buchs, Switzerland) during a 45-minute incubation step in Dulbecco modified Eagle medium (Invitrogen, Zug, Switzerland) with 10% FCS (Sigma, Buchs, Switzerland) at 37°C/5% CO_2 _[26]. The epithelial organoids were directly processed to single-cell suspensions by digestion in Hyclone HyQTase (ThermoScientific, Lausanne, Switzerland) with gentle pipetting for 15 minutes at 37°C. The cell suspension was filtered through a 40-μm cell strainer (BD Falcon, Basel, Switzerland), and the final cell suspension pelleted at 650 *g *for 4 minutes.

### Cell labeling and flow cytometry

**Figure 1:**
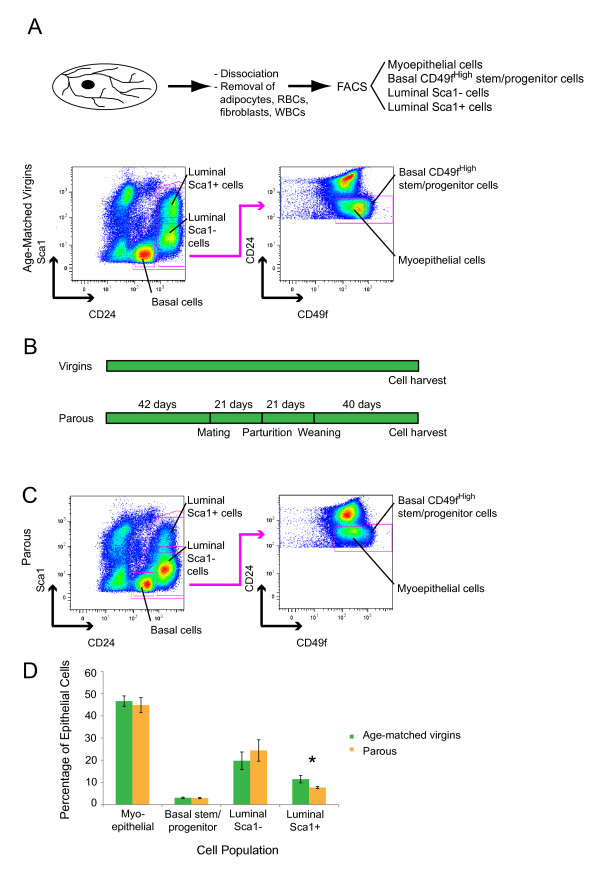
**The CD24/Sca1 and CD49f^High^/CD24 flow-cytometry profiles of parous and age-matched virgin mice are similar**. **(A) **Schematic illustration of the cell-isolation strategy and representative flow-cytometry pseudocolor plots of mammary cells from age-matched virgin control mice. After depletion of CD45^+ ^white blood cells, luminal and basal mammary epithelial cells were separated on the basis of CD24 and Sca1 expression. Further separation of basal cells into myoepithelial and basal stem/progenitor cell subpopulations was based on the expression of CD24 and CD49f. The isolated mammary epithelial cell subpopulations included luminal Sca1^+ ^(CD24^+High^Sca1^+^) cells, luminal Sca1^- ^(CD24^+High^Sca1^-^) cells, basal CD49f^High ^(CD24^+Low^Sca1^-^CD49f^High^) or basal stem/progenitor cells, and basal myoepithelial (CD24^+Low^Sca1^-^CD49f^Low^) cells. **(B) **Outline of the mouse mating, parturition, weaning, and involution protocol. **(C) **Representative flow-cytometry pseudocolor plots of mammary cells from parous mice. The gates applied were the same as those for age-matched virgin controls. **(D) **Bar graph showing the distribution of mammary epithelial cell subpopulations comparing cells from parous with age-matched virgin control mice. Data represent the mean ± SEM of seven cell-isolation experiments with a minimum of 10 mice per experiment. The proportion of luminal Sca1^+ ^cells was reduced by approximately 50% in parous mice (*P *= 0.02 with a two-tailed unpaired Student *t *test).

Cells were labeled as previously described [26] by using the antibodies PE-Cy7-CD45 (1:33), FITC-CD24 (1:40), PE-CD49f (1:40), and APC-Sca1 (1:40). Detailed antibody information is given later. DAPI (0.2%, Invitrogen, Zug, Switzerland) was added 10 minutes before cell sorting (1:250). FACS was carried out on a MoFlo cell sorter (Becton Dickinson, Basel, Switzerland). Cells were gated based on their forward- and side-scatter profiles (FS Area and SS Area). A time-of-flight approach (pulse width) was used to exclude doublets and higher-order cell clumps. Dead cells (DAPI bright) and immune cells (CD45^+^) were gated out (see Additional file 1). The gate for basal stem/progenitor cells was set at the top 5% of CD49f-expressing cells, as described [19,21] (see Figure 1). Routine examination of the sorted mammary epithelial cell subpopulations showed a degree of purification higher than 95%.

### *In vitro *colony formation assay and quantification

Freshly sorted cells of each subpopulation (500 cells) were plated onto irradiated 3T3L1 feeder cells (5,000 cells) in 24-well Primaria plates (Becton Dickinson, Basel, Switzerland). The cells were cultured in DMEM/Ham F12 (Invitrogen, Zug, Switzerland) with 10% FCS, 100 IU/ml penicillin, 100 μg/ml streptomycin (Invitrogen, Zug, Switzerland), 5 μg/ml bovine pancreatic insulin (Sigma, cell culture-tested solution, Buchs, Switzerland), 10 ng/ml mouse EGF (BD Biosciences, Basel, Switzerland), and 10 ng/ml cholera toxin (Sigma, Buchs, Switzerland). After 24 hours, the medium was renewed, and 4 days later, the colonies were fixed with acetone/methanol (1:1), washed, and rehydrated with PBS and 0.05% NaN_3_. In separate rescue experiments with selected mammary epithelial cells from parous mice, the incubation medium was supplemented with recombinant mouse Wnt4 (R&D Systems, Abingdon, UK) at 500 ng/ml. For quantification, colonies were immunofluorescently stained with Krt18 and Krt14 antibodies and with Hoechst 33342. The stained colonies were imaged with a MacroFluo Z16 microscope (Leica, Heerbrugg, Switzerland) at 2× magnification, and a Z1 microscope (Zeiss, Feldbach, Switzerland) at 5× magnification. The feeder cells served as negative controls for Krt18 and Krt14 staining. The number of colonies (that is, clusters of more than three cells [23]) per well was determined manually. Colonies defined as Krt18/Krt14 double-positive were double positive over a minimum of 20% of colony area.

### Mammary fat pad transplantation

Freshly sorted basal stem/progenitor cells were resuspended in PBS with 50% Matrigel (BD Biosciences, Basel, Switzerland) and injected (30 μl) in limiting dilution numbers into the cleared fourth mammary glands of 3-week-old syngeneic female mice. Glands from recipient mice were harvested 8 to 9 weeks after transplantation, processed as whole mounts (see earlier), and scanned with an Epson Expression 1600 Pro scanner. The number of outgrowths was counted, with an outgrowth defined as an epithelial structure with ducts starting from a central point and with lobules and/or terminal end buds (see Additional file 2) [27].

### Microarray analyses

**Figure 2:**
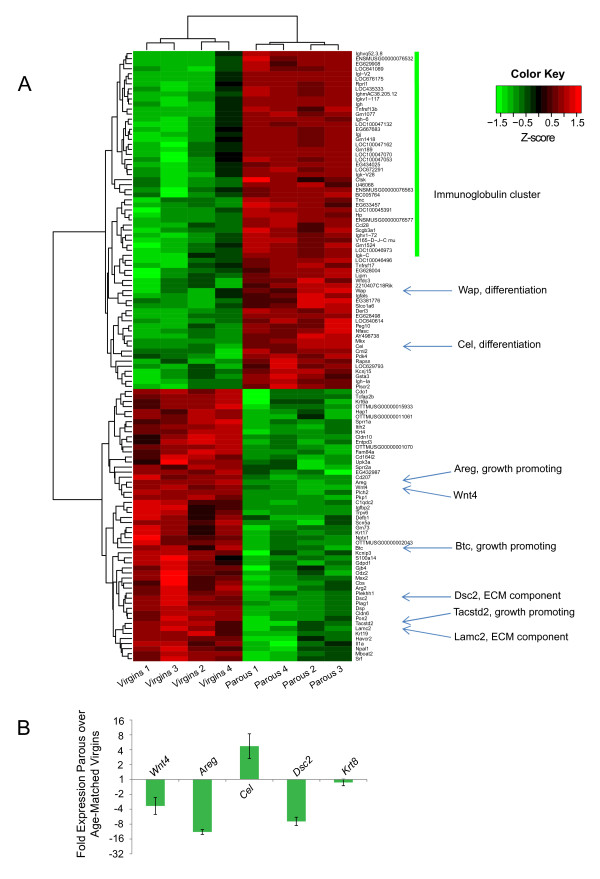
**Parity-induced gene signature in total mammary cell suspensions**. **(A) **Heat-map and cluster analysis of differential gene expression in total isolated mammary cells from age-matched virgin control and parous mice. Gene expression is presented as normalized Z-scores, defined as Z = (x-μ)/sd to allow visualization. A cut-off of *P *< 0.05 was applied. If multiple probe sets existed for the same gene, the probe set with the largest change in expression was selected. Four independent experiments were performed with 10 mice (five virgins; five parous) per experiment. **(B) **qPCR validation of pregnancy-induced gene-expression changes in total mammary cell suspensions. Fold changes are shown in relation to expression in cell suspensions from age-matched virgin control mice. Ct values were normalized to the reference gene *Hprt*. As control for luminal epithelial cell number, qPCR for *Krt8 *was performed. Data represent the mean ± SEM of four independent experiments with 10 mice (five virgins; five parous) per experiment.

**Figure 3:**
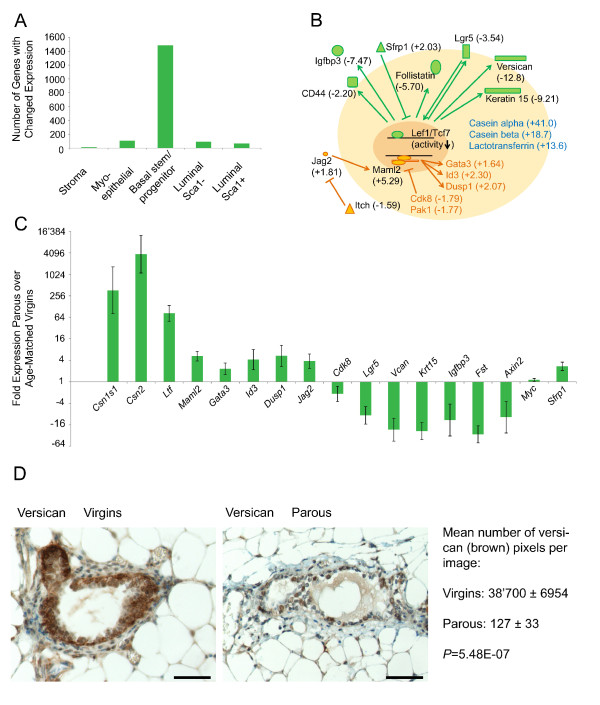
**Parity leads to differentiation and decreases the Wnt/Notch signaling ratio in basal stem/progenitor cells**. **(A) **Bar graph depicting the number of gene-expression changes in FACS-sorted mammary stromal and epithelial cell subpopulations from parous mice compared with age-matched virgin control mice by using a cut-off of fold change > 1.5 and an adjusted *P *value < 0.05. By far the most gene-expression changes were observed in basal stem/progenitor cells. Three independent experiments were performed with 10 mice (five virgins; five parous) per experiment. **(B) **Schematic illustration of prominent gene-expression changes in FACS-sorted basal stem/progenitor cells from parous as compared with age-matched virgin control mice. Fold changes are shown in parentheses with upregulated genes denoted as positive (+), and downregulated genes, as negative (-). Differentiation genes were upregulated (blue), Wnt target genes were downregulated (green), Wnt inhibitor *Sfrp1 *was upregulated (green) and overall Notch signaling (orange) was increased in basal stem/progenitor cells from parous mice. **(C) **qPCR validation of the changes in gene expression in basal stem/progenitor cells of parous mice. All classic Wnt target genes were downregulated, including *Lgr5, Axin2*, and versican (*Vcan*), whereas the more ubiquitously regulated target *Myc *was unchanged. In all cases, fold changes are shown relative to cells from age-matched virgin control mice. Ct values were normalized to the reference genes *Hprt *and *Ubc *[63]. Data represent the mean ± SEM of three independent experiments with 10 mice (five virgins; five parous) per experiment. **(D) **Representative images and quantification of immunostaining for the Wnt target gene versican in mammary gland sections from age-matched virgin and parous mice in estrus. Scale bar, 50 μm. Quantitative data represent the mean ± SEM from 60 randomly selected images from three virgin and three parous mice.

**Figure 4:**
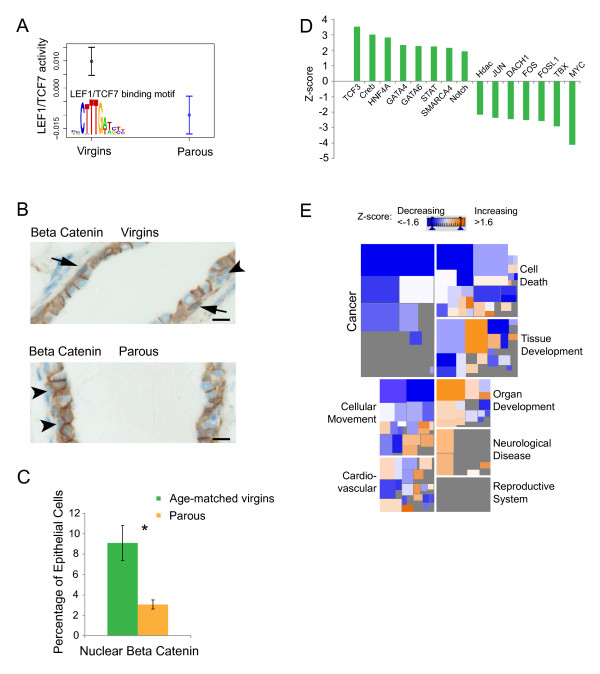
**Effects of parity on Wnt transcription-factor activities and nuclear β-catenin in basal mammary epithelial cells**. **(A) **Wnt transcription factor LEF1/TCF7 motif activity in basal stem/progenitor cells from parous as compared with virgin control mice, as predicted by MARA [32]. The binding motif of the LEF1/TCF7 transcription factor is shown in color. **(B) **Representative images of immunostaining for β-catenin in mammary gland sections from age-matched virgin and parous mice in estrus. Arrow, basal mammary epithelial cells with nuclear β-catenin. Arrowhead, basal mammary epithelial cells lacking nuclear β-catenin. Scale bar, 10 μm. **(C) **Bar graph representing the quantification of nuclear β-catenin in mammary gland sections of virgin and parous mice. Data represent the mean ± SD (virgin mice: *n *= 3; parous mice: *n *= 3). *P *= 0.004 with the two-tailed unpaired Student *t *test. **(D) **Transcription-factor activities in basal stem/progenitor cells, predicted on the basis of target gene expression by Ingenuity IPA [40]. Activity is reported as Z scores (positive Z score, upregulation; negative Z score, downregulation) by using a cut-off of linear fold change > 1.5 and *P *value < 0.05. **(E) **BioFunctions most strongly downregulated (blue) or upregulated (orange) in basal stem/progenitor cells, as calculated by Ingenuity IPA [40]. The color of the fields represents the Z score; the size of the fields represents the number of genes. A stringent cut-off of linear fold change > 2 and *P *value < 0.01 was used to minimize the number of false positives.

Microarray analyses were performed on unsorted total mammary cell suspensions (see Figure 2) and on FACS-sorted mammary cell subpopulations (see Figures 3 and 4, as well as Table 1). For unsorted mammary cells, RNA was extracted by using the RNeasy Plus Mini Kit (Qiagen, Hilden, Germany). Genomic DNA was removed by using gDNA Eliminator Mini Spin Columns (Qiagen, Hilden, Germany). The RNA concentration was measured with a Nanodrop 1000 machine, and RNA quality determined with an Agilent 2100 bioanalyzer and RNA Nano Chips. Aliquots of 100 ng of isolated total RNA were amplified by using the Ambion WT Expression kit (Ambion, Zug, Switzerland). For FACS-sorted mammary epithelial cell subpopulations, total RNA was isolated by using the Arcturus PicoPure Frozen RNA Isolation Kit (Life Technologies, Zug, Switzerland). Genomic DNA was removed by using RNase-Free DNase (Qiagen, Hilden, Germany), the RNA concentration determined by using the RiboGreen Assay, and RNA quality assessed by using an Agilent 2100 bioanalyzer and RNA Pico Chips. Total RNA was used as the input for synthesis of amplified cDNA with the NuGen Ovation Pico WTA System (NuGen Inc., Leek, The Netherlands).

**Table 1:** Wnt signaling is decreased and Notch signaling is upregulated in basal stem/progenitor cells

	Wnt signaling				Notch signaling			
	
	NES	*P *value	FDR	FWER	NES	*P *value	FDR	FWER
Myoepithelial	-1.47	0.03	0.06	0.04	1.08	0.35	0.34	0.74
**Basal stem/progenitor**	**-1.70**	**< 0.01**	**< 0.01**	**< 0.01**	**1.64**	**< 0.01**	**0.02**	**0.01**
Luminal Sca1^-^	-1.08	0.33	0.64	0.37	1.08	0.32	0.62	0.45
Luminal Sca1^+^	-0.68	0.99	0.99	0.74	0.83	0.77	1.00	0.93

Gene-set enrichment analysis (GSEA) for Wnt and Notch signaling, as defined by the expression of Wnt or Notch target genes (see Methods) showed a decrease in the Wnt/Notch signaling ratio (strongly negative/positive normalized enrichment scores (NES)), which was specific for the basal stem/progenitor cells. Significance was determined by a cut-off of a nominal *P *value < 0.01, a false-discovery rate (FDR) < 0.05, and a family-wise error rate (FWER) < 0.05, as suggested by the GSEA homepage [28]. The gene set for Wnt signaling consisted of the reported Wnt targets in mammalian systems (derived from the Stanford Wnt homepage) and is depicted in Additional file 3. The gene set for Notch signaling was derived from the Broad Institute v3.0 and is termed Nguyen Notch1 targets. The number of permutations was set to 1,000, and the permutation type was set to "gene set," because fewer than seven samples existed per phenotype [28]. Otherwise, the default settings were used.

Resulting double-stranded cDNA was fragmented and labeled by using the Affymetrix GeneChip WT Terminal Labeling kit (Affymetrix, High Wycombe, UK). Affymetrix Gene Chip Mouse gene 1.0 ST microarrays were hybridized according to the GeneChip Whole Transcript (WT) Sense Target Labeling Assay Manual (Affymetrix, High Wycombe, UK) with a hybridization time of 16 hours. The Affymetrix Fluidics protocol FS450_0007 was used for washing. Scanning was performed with Affymetrix GCC Scan Control Software v. 3.0.0.1214 on a GeneChip Scanner 3000 7 G with autoloader. Arrays were normalized, and probeset-level expression values calculated with R/Bioconductor's (v2.14) "affy" package by using the rma() function. Differential gene expression between experimental and control samples was determined by using linear modeling as implemented in the R/Bioconductor package "limma." For general analysis of gene expression in total mammary cell suspensions and in FACS-sorted mammary cell subpopulations, we used the cut-off linear fold change > 1.5, adjusted *P *value < 0.05, and average linear expression between conditions greater than 4. To determine the 10 most up- or downregulated genes in FACS-sorted mammary epithelial cell subpopulations, a cut-off of linear fold change > 2.0, adjusted *P *value < 0.001, and average linear expression between conditions ave > 4 was used. For FACS-sorted mammary epithelial cell subpopulations both resulting lists of differential genes were imported into Ingenuity IPA (Ingenuity, content version 12710793) for pathway analysis. Gene-set enrichments in FACS-sorted mammary epithelial cell subpopulations were determined by using the JAVA application from the Broad Institute v2.0 [28,29] and gene sets v2.5 and v3.0, as well as custom gene sets (see Additional file 3).

The microarray data from this publication have been submitted to the NCBI Gene Expression Omnibus [30] and are deposited as GSE40875 (mouse mammary cell subtypes), GSE40876 (total mammary epithelial cells in mice), and GSE40877 (both total mammary epithelial cells and mammary cell subtypes in mice).

### Quantitative PCR

RNA was isolated as described earlier and converted to cDNA by using the WT-Ovation Exon Module Version 1.0 (NuGen Inc., Leek, The Netherlands). Real-time PCR was performed on the unamplified cDNA corresponding to the specified number of cells or on 25 ng of amplified cDNA (by using the NuGen Ovation Pico WTA System (NuGen Inc., Leek, The Netherlands) for amplification). The TaqMan probe-based system was applied in combination with the TaqMan Universal PCR Master Mix (Applied Biosystems, Zug, Switzerland). The probe IDs are given later. Cycling was performed with 7500 Fast and Step OnePlus Real-Time PCR Systems (both from Applied Biosystems, Zug, Switzerland).

### Probe IDs for quantitative PCR

These were the probes: Areg (Mm00437583_m1), Axin2 (Mm00443610_m1), B2M (Mm00437762_m1), Cdk8 (Mm01223097_m1), Cel (Mm00486975_m1), Csn1s1 (Mm00514430_m1), Csn2 (Mm04207885_m1), Dsc2 (Mm00516355_m1), Dusp1 (Mm00457274_g1), Esr1 (Mm00433149_m1), Fst (Mm00514982_m1), Gata3 (Mm00484683_m1), Hprt (Mm00446968_m1), Id3 (Mm00492575_m1), Igfbp3 (Mm_01187817_m1), Jag2 (Mm01325629_m1), Krt8 (Mm00835759_m1), Krt14 (Mm00516876_m1), Krt15 (Mm00492972_m1), Krt19 (Mm00492980_m1), Lgr5 (Mm00438890_m1), Ltf (Mm00434787_m1), Maml2 (Mm00620617_m1), Myc (Mm00487804_m1), Pgr (Mm00435628_m1), Sfrp1 (Mm00489161_m1), Ubc (Mm01201237_m1), Vcan (Mm01283063_m1), Wnt4 (Mm01194003_m1).

### Immunofluorescent staining

For single-cell staining, freshly sorted cells were allowed to air-dry on poly-L-lysine-coated slides and stored at -20°C. The dried cells were blocked with PBS, 2.5% goat serum, and 0.05% NaN_3 _for 60 minutes under UV light. The UV treatment was used to attenuate residual fluorescence from bound FACS antibodies. Primary antibody staining was performed overnight at 4°C by using Krt18 antibody (1:1,000) and Krt14 antibody (1:500) as luminal and basal cell markers, respectively. Secondary antibody staining was carried out for 60 minutes at room temperature by using anti-guinea pig Ig-Alexa488 (1:500) and anti-rabbit Ig-Alexa546 (1:1,000). Hoechst 33342 (0.5 μg/ml; Invitrogen, Zug, Switzerland) staining was performed for 10 minutes at room temperature. The stained cells were mounted with ProLong Gold antifade reagent (Invitrogen, Zug, Switzerland) and imaged with a Z1 microscope (Zeiss, Feldbach, Switzerland) at 63× magnification. No primary antibody was added as a negative control.

### Immunohistochemistry

The fourth mammary glands of parous or age-matched virgin control mice were collected 40 days after weaning from mice in estrus. The glands were fixed in 4% PFA and embedded in paraffin. For immunostaining with ERα and PR, the sections were dewaxed and subjected to antigen retrieval by boiling in 10 m*M *citrate buffer for 10 minutes. Subsequently, the sections were cooled to room temperature, quenched for 10 minutes with PBS and 3% H_2_O_2_, and blocked for 30 to 60 minutes with PBS and 2.5% NGS. ERα and PR primary antibody staining was performed overnight at 4°C at a 1:1,000 (ERα) and a 1:200 (PR) dilution. Secondary antibody staining was carried out for 30 to 60 minutes at room temperature with biotinylated anti-rabbit IgG. Immunohistochemistry for versican, β-catenin, and p21 was performed on the Ventana DiscoveryXT instrument (Roche Diagnostics, Rotkreuz, Switzerland) by using the Research IHC Dap Map XT procedure. In brief, dewaxing was performed in the machine, and slides were pretreated with mildCC1 (versican and p21) or standardCC1 (β-catenin) (Roche Diagnostics, Rotkreuz, Switzerland). Primary antibodies were incubated at 37°C for 1 hour at the following dilutions: versican, 1:50; p21, 1:50; and β-catenin, 1:500. As secondary antibody, Immpress, an anti-rabbit HRP conjugated polymer, was used. All slides were counterstained with Hematoxylin II (Roche Diagnostics, Rotkreuz, Switzerland) and Bluing Reagent (Roche Diagnostics, Rotkreuz, Switzerland). Images were captured at 20-fold (ERα or PR) or 40-fold (versican, β-catenin, p21) magnification by using an Eclipse E600 microscope (Nikon, Egg, Switzerland). For quantification of ERα and PR positivity, at least 2,000 epithelial cells per mouse were counted. For quantification of nuclear β-catenin and p21, at least 500 epithelial cells per mouse were counted. The quantification of versican was performed with the MATLAB software by using color segmentation based on Mahalanobis distance to determine the pixels with a particular RGB-color distribution [31].

### Determination of blood progesterone concentrations

Blood was collected from the right atrium of mice in estrus in EDTA-covered tubes (Sarstedt, Numbrecht-Rommelsdorf, Germany), and plasma was extracted by centrifugation at 1,500 *g *for 15 minutes at 4°C. Progesterone concentrations were assessed with ELISA, as specified by the manufacturer's guidelines (DRG, catalog no. EIA-1561, Marburg, Germany).

### Antibodies

For flow cytometry, the following anti-mouse antibodies were used: PE-Cy7-CD45 (clone 30-F11), FITC-CD24 (clone M1/69), PE-CD49f (clone GoH3) (all from BD Pharmingen, Basel, Switzerland), and APC-Sca1 (clone E13-161.7, from Biolegend, San Diego, USA).

For immunofluorescence staining, the primary anti-mouse antibodies used were guinea pig keratin 18 (Krt18; Fitzgerald; catalog no. GP11, North Acton, USA) and rabbit keratin 14 (Krt14; ThermoScientific, catalog no. RB-9020, Lausanne, Switzerland). The secondary antibodies used were anti-guinea pig Ig-Alexa488 and anti-rabbit Ig-Alexa546 (Molecular Probe, Invitrogen, Zug, Switzerland).

For immunohistochemistry staining, the primary antibodies included estrogen receptor alpha (ERα; Santa Cruz Biotechnology; catalog no. SC-542, Dallas, USA), progesterone receptor (PR; Clone SP2, ThermoScientific; catalog no. RM-9102, Lausanne, Switzerland), versican (Millipore/Chemicon; catalog no. ab1033, Billerica, USA), β-catenin (Cell Signaling; catalog no. 9587, Danvers, USA), and p21 (Abcam; catalog no. ab2961, Cambridge, UK). The secondary antibodies used were biotinylated anti-rabbit IgG (Vector Labs; catalog no. BA-1000, Petersborough, UK) and anti-rabbit HRP conjugated polymer Immpress (Vector Labs; catalog no. MP-7401, Petersborough, UK).

### Statistics

The limited dilution transplantation data were analyzed statistically as published previously [18]. The two-tailed unpaired Student *t *test was used to determine statistical significance of comparisons.

### Motif Activity Response Analysis (MARA)

The MARA model [32] combines knowledge of gene-expression levels (measured by microarray) with transcription-factor binding sites to answer the question of which transcription factors are driving expression changes in mammary stem/progenitor cells in parous as compared with age-matched virgin control mice. Specifically, log-expression levels of all genes present on the microarray were modeled as linear combinations of transcription factor activities. The coefficients of these combinations were determined by the number of transcription-factor binding sites in the proximal promoter regions. For each transcription-factor binding motif *m *and each sample (microarray) *s*, we estimated the activity A*_ms _*with the corresponding error. Furthermore, we quantified the significance of activity change of each binding motif in parous as compared with virgin control mice.

## Results

### Early pregnancy decreases luminal Sca1^+ ^cells, but does not change the proportions of the other mammary epithelial cell subpopulations

To investigate the influence of early parity on the proportions of mammary epithelial cell subtypes, we first established FACS profiles of epithelial cell subpopulations in virgin FVB control mice [21,26]. Luminal CD24^+High ^Sca1^+ ^cells, luminal CD24^+High ^Sca1^- ^cells, basal CD24^+Low ^Sca1^- ^CD49f^High ^cells, and basal CD24^+Low ^Sca1^- ^CD49f^Low ^myoepithelial cells were isolated from these mice (Figure 1A). Use of the established cell markers keratin 18 (Krt18) and keratin 14 (Krt14) confirmed the luminal and basal origin of the isolated cell subpopulations, and qPCR for *CD49f *and *Sca1 *affirmed the purity of the isolated cell subpopulations (see Additional file 4). Because basal CD49f^High ^cells are considered to be enriched for basal mammary stem/progenitor cells in virgin mice [19,21], the term "basal CD49f^High ^cells" is used synonymously with "basal stem/progenitor cells" throughout this article. To extend these analyses to parous mice, we confirmed that involution was complete 28 and 40 days after weaning (see Additional file 5). To allow a margin of safety, 40 days after weaning was used for all subsequent cell-isolation experiments in a standardized parturition protocol with mating at 42 days (Figure 1B). The FACS profiles of epithelial cell subpopulations from parous mice and age-matched virgin control mice were similar (Figure 1C, D; Additional file 1) with the exception of luminal Sca1^+ ^cells, which decreased by about 50% in parous mice (*P *= 0.02). Of note, luminal Sca1^+ ^cells have been shown to be enriched for hormone receptor-positive cells [17]. This was verified by qPCR (see Additional file 6). These data demonstrate that the adopted experimental procedure permits the isolation of all epithelial cell subpopulations from parous mice, including basal stem/progenitor cells, at levels adequate for transcriptomic and functional analyses.

### Parity upregulates differentiation genes in all cell subpopulations and decreases the Wnt/Notch signaling ratio in the basal stem/progenitor cell subpopulation

Next we investigated the effects of early pregnancy on the gene-expression profiles of the isolated mammary epithelial cell subpopulations. To control for the effect of the cell-isolation procedure on gene expression, we performed first a transcriptome and cluster analysis in non-FACS-sorted total mammary cell suspensions from age-matched virgin and parous mice (Figure 2A). The analysis showed that pregnancy induces an upregulation of many immunoglobulin and differentiation genes (for example, whey acidic protein *Wap*, and carboxyl ester lipase *Cel*) and a downregulation of growth factors (for example, amphiregulin *Areg*, betacellulin *Btc*, tumor-associated calcium signal transducer 2 *Tacstd2*), and extracellular matrix (ECM) elements (for example, laminin, gamma 2 *Lamc2*, desmocollin 2 *Dsc2*). These data are consistent with the published pregnancy-induced gene signature determined in snap-frozen rodent mammary glands [9,10]. Thus, the gene signature was not lost during the isolation procedure, confirming the validity of our experimental system. Furthermore and most interestingly, a novel 3.4-fold downregulation of the Wnt signaling protein Wnt4 was observed in cells from parous mice (Figure 2). These microarray data were validated by qPCR for four genes, including *Wnt4*. Importantly, the expression of the luminal marker *Krt8 *was not altered on parity, demonstrating that the observed changes in gene expression were independent of unspecific alterations in total luminal cell numbers (Figure 2B).

Because the number of isolated basal stem/progenitor cells for gene-profiling analysis was limited compared with the other cell subpopulations (that is, myoepithelial, luminal Sca1^-^, and luminal Sca1^+ ^cells), it was important to evaluate the influence of different cell numbers on transcriptome analysis. As shown in Additional Material, we found that it is valid to use cell numbers in the range of 2,000 to 50,000 cells for comparison of transcriptomes from different mammary cell subpopulations (Additional file 6). Furthermore, qPCR analysis of nonamplified and amplified cDNA for known basal (*Krt14*) and luminal (*Krt8*; *Krt19*) cell marker genes confirmed that the amplification process of the microarray analysis was unbiased (Additional file 6).

In the subsequent transcriptome analysis of FACS-sorted mammary epithelial cell subpopulations from age-matched virgin control and parous mice, all mammary epithelial cell subpopulations, except immune cell-depleted stromal cells, showed parity-induced changes in gene expression with by far the most prominent effects being scored for basal stem/progenitor cells (Figure 3A). Furthermore, although differentiation genes were upregulated in all epithelial cell subpopulations, the strongest prodifferentiation effects were seen in basal stem/progenitor cells from parous mice. For example, casein alpha 1 (*Csn1s1*), casein beta (*Csn2*), and lactotransferrin (*Ltf*) were upregulated 41-, 19-, and 14-fold, respectively (Figure 3B). Apart from these differentiation genes, the Notch co-activator *Maml2 *was found among the 10 most-upregulated genes (by fold change and/or by *P *value) in basal stem/progenitor cells from parous mice. In contrast, the 10 most-downregulated genes included the Wnt target and co-receptor *Lgr5*, the Wnt target and epithelial stem cell marker keratin 15 (*Krt15*) [33], and the Wnt targets versican (*Vcan*) and *Igfbp3 *[34] (Figure 3B). Extension of the analysis to all data for signaling-pathway genes revealed further Wnt target genes, which were downregulated (for example, *CD44 *and follistatin (*Fst*) [35,36]), and the Wnt inhibitor *Sfrp1*, which was upregulated in basal stem/progenitor cells from parous mice. Moreover, in the same epithelial cell subpopulation of parous mice, the Notch ligand *Jag2 *and the Notch target genes *Gata3, Id3*, and *Dusp1 *were upregulated, whereas the Notch inhibitor *Itch *and the Maml2 RBP-J complex inhibitors *Cdk8 *and *Pak1 *[37] were downregulated (Figure 3B). These gene-expression profiling data were validated with qPCR for several selected genes (Figure 3C). Furthermore, one of the classic Wnt target genes (versican) was examined on the protein level and found to be strongly downregulated in the basal compartment of mammary glands from parous mice (Figure 3D). Thus, the data demonstrate strong upregulation of differentiation genes, a downregulation of Wnt target genes, and an increase in Notch signaling in the basal stem/progenitor-cell subpopulation of parous mice.

Further verification of these conclusions came from Motif Activity Response Analysis (MARA) of transcription factor activities (see Methods) [38]. The canonical Wnt transcription factor LEF1/TCF7 was shown to have significantly decreased activity in basal stem/progenitor cells from parous mice (Figure 4A), which is consistent with the observed decrease in canonical Wnt signaling in the previous gene-expression profile analysis.

Final confirmation of parity-induced downregulation of Wnt signaling in mammary glands was provided by immunohistochemical staining of mammary gland sections from age-matched virgin control and parous mice for β-catenin. As illustrated in Figure 4B, nuclear β-catenin was observed in basal but not in luminal mammary epithelial cells of virgin mice. In parous mice, the proportion of basal mammary epithelial cells positive for nuclear β-catenin was significantly decreased (Figure 4C). Because nuclear β-catenin is necessary for Wnt target gene expression, this finding represents an additional verification of parity-induced downregulation of Wnt signaling (Figure 3).

### The parity-induced decrease in Wnt/Notch signaling ratio is specific for the basal stem/progenitor cell subpopulation

To assess whether the observed decrease in canonical Wnt and increase in Notch signaling were specific for basal stem/progenitor cells, we next investigated the enrichment of Wnt/Notch signaling genes over all genes altered in the various FACS-sorted mammary epithelial cell subpopulations from parous as compared with virgin control mice. Such analysis showed that canonical Wnt signaling was significantly downregulated in basal stem/progenitor but not in luminal Sca1^- ^or luminal Sca1^+ ^cells from parous mice (Table 1; see Additional file 7). In myoepithelial cells, a trend toward a decrease in Wnt signaling was not significant when applying a very stringent cut-off (see Table 1 legend) and was probably due to contamination of the myoepithelial cell subpopulation with basal stem and/or progenitor cells. Similarly, Notch signaling was found to be significantly upregulated in basal stem/progenitor cells but in no other mammary epithelial cell subpopulation (Table 1). Whereas downregulation of Wnt and upregulation of Notch signaling were specific for basal stem/progenitor cells, similar enrichment analyses for genes involved in other signaling pathways revealed that the previously reported upregulation of the p53-p21 pathway [39] occurred in all epithelial cell subpopulations tested (Additional file 7).

Further bioinformatics analysis of the data with different software [40] provided a second line of verification of the specific decrease in Wnt signaling in basal stem/progenitor cells after parity. Performing transcription-factor activity analyses based on target gene expression, we found TCF3, an inhibitor of the canonical Wnt signaling pathway [41], to be the transcription factor with the highest z-score (*Z *= 3.521) and the protooncogene MYC to have a very low z-score (*Z *= -4.108) in basal stem/progenitor cells but not in other mammary epithelial cell subpopulations of parous mice (Figure 4D, z-score defined as (x-μ)/sd and used as a measure for transcription factor activity based on the expression levels of target genes). These findings confirm the results obtained in the MARA analysis. Furthermore, inhibition of MYC leads to upregulation of *Sfrp1*, which in turn inhibits canonical Wnt signaling [42]. Upregulation of *Sfrp1 *was observed in the microarray analysis and validated with qPCR (Figure 3B, C), thus directly reflecting the expected effects of the bioinformatic predictions.

Downregulation of canonical Wnt signaling and MYC activity would be expected to decrease the propensity for cancer, and indeed, in an analysis of biofunctions, a marked and consistent decrease in cancer-associated functions, was observed for basal stem/progenitor cells (Figure 4E), but no other mammary epithelial cell subpopulation. This potential anticancer phenotype of basal stem/progenitor cells was underscored by gene-enrichment analyses on all available pathway gene sets (see Additional file 8), which showed a strong downregulation of proliferation- and tumorigenesis-associated gene sets.

### Parity decreases the *in vitro *clonogenic potential to the greatest extent in the basal stem/progenitor cell subpopulation

**Figure 5:**
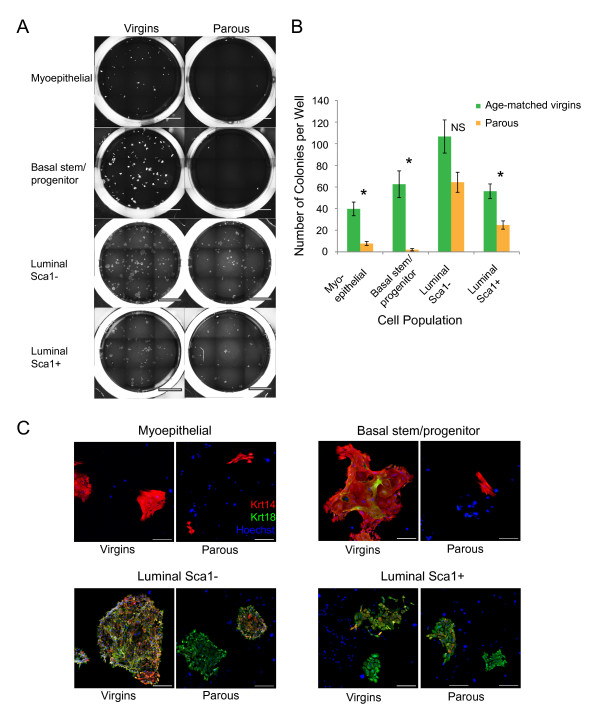
**Parity reduces the progenitor potential of mammary epithelial cell subpopulations**. **(A) **Representative images of individual wells with colonies formed by the specified cell subpopulations from age-matched virgin and parous mice. Scale bar, 4 mm. **(B) **Bar graph comparing the colony-forming capacities of myoepithelial cells, basal stem/progenitor cells, luminal Sca1^-^, and luminal Sca1^+ ^cells of age-matched virgin and parous mice. Data are from three independent experiments and represent the mean ± SEM of colonies per well; 18 wells were assessed per cell type. *P < 0.015, NS: not significant (*P *= 0.08), by using two-tailed unpaired Student *t *test. **(C) **Representative images for the immunophenotyping of 5-day-old colonies grown from myoepithelial cells, basal stem/progenitor cells, luminal Sca1^-^, and luminal Sca1^+ ^cells. The colonies were stained for luminal Krt18 (green) and basal Krt14 (red) expression. Hoechst 33342 (blue) was used to distinguish nuclei and to label feeder cells (negative control). Scale bar, 200 μm.

Because decreased Wnt signaling and increased Notch signaling have been shown to decrease *in vitro *and *in vivo *proliferation of basal stem/progenitor cells [43,44], we next assessed the *in vitro *colony-formation capacities of mammary epithelial cell subpopulations from parous and age-matched virgin mice (Figure 5A). In virgin control mice, luminal Sca1^- ^cells had the highest colony-formation capacity, with an average of 107 colonies per well (Figure 5B). This strong clonogenic potential suggests a pronounced progenitor identity of luminal Sca1^- ^cells and is consistent with previous observations in younger virgin mice [16,17]. A high colony-formation capacity was also observed for the basal stem/progenitor cells of virgin mice (63 colonies per well) (Figure 5B), which is consistent with the notion that CD49f^High ^cells contain a high proportion of basal progenitor cells as well as putative mammary stem cells [45]. With the exception of luminal Sca1^- ^cells, the colony-formation capacities of all epithelial cell subpopulations were lower in parous mice than in age-matched virgins (Figure 5B). Thereby, by far the most pronounced difference was observed in basal stem/progenitor cells (Figure 5B). A substantial decrease in the colony-formation capacity was also seen for the myoepithelial cell subpopulation, which also contains basal progenitor cells. Of note, basal stem/progenitor cells from parous mice did not die but remained as quiescent single cells or divided only once during 5 days of culture (Figure 5C).

The reduced progenitor potential of basal mammary epithelial cell subtypes and especially of basal stem/progenitor cells from parous mice was further confirmed by phenotypic analysis of colonies by colony size (cell number) and immunophenotyping of luminal and basal markers Krt18 and Krt14, respectively. The largest difference in colony size was observed for the basal stem/progenitor cell subpopulation, where parity induced a decrease in colonies of ≥ 20 cells from 66% to 9% (Table 2A; Figure 5C). Considerably smaller parity-induced reductions in colony size were observed for the other epithelial cell subpopulations, although a substantial decrease was also seen for basal myoepithelial cells (Table 2A). With regard to Krt18/Krt14 double positivity, parity induced a reduction in Krt18/Krt14 double-positive colonies derived from basal stem/progenitor cells from 26% to 0 (Table 2A). In contrast, although Krt18/Krt14 double-positivity decreased by 27%, 57% of colonies derived from luminal Sca1^- ^cells of parous mice maintained double positivity (Table 2A). No effects of parity on Krt18/Krt14 double positivity were observed for colonies derived from luminal Sca1^+ ^or basal myoepithelial cells (Table 2A), whereby myoepithelial cells did not give rise to double-positive colonies even when originating from virgin control mice. Thus, consistent with the basal mammary stem/progenitor cell subpopulation-specific reduction in the Wnt/Notch signaling ratio, parity decreased colony-formation capacity, *in vitro *proliferation potential (colony number and size), and Krt18/Krt14 double positivity most prominently in the basal stem/progenitor cell subpopulation.

**Table 2:** Functional characterization of isolated epithelial cell subpopulations

A	Fraction of large colonies			Fraction of double-positive colonies		
	
	Virgins		Parous	Virgins		Parous
Myoepithelial^a^	32.7 ± 7.4%^a^		9.6 ± 2.3%^a^	0%		0%

Basal stem/progenitor^a^	66.2 ± 7.2%^a^		9.3 ± 4.5%^a^	26.1 ± 2.7%		0%

Luminal Sca1^-b^	36.3 ± 5.6%^b^		31.3 ± 2.6%^b^	84.0 ± 3.4%		57.1 ± 2.9%

Luminal Sca1^+a^	73.3 ± 1.8%^a^		71.8 ± 4.1%^a^	64.1 ± 3.6%		59.8 ± 2.7%

**B** **Number of basal stem/progenitor cells injected per cleared fat pad**	**Number of positive outgrowths**^**c, d, e**^					
	
	**Age-matched virgin control donors**			**Parous donors**		

1,000	^c^8/9	^d^8/9	^e^8/9	^c^3/9	^d^3/9	^e^4/9

500	^c^8/11	^d^8/11	^e^8/11	^c^2/11	^d^2/11	^e^6/11

250	^c^2/6	^d^2/6	^e^2/6	^c^0/6	^d^1/6	^e^1/6

100	^c^0/6	^d^1/6	^e^1/6	^c^0/6	^d^0/6	^e^2/6

50	^c^0/7	^d^0/7	^e^0/7	^c^1/7	^d^1/7	^e^1/7

Repopulating frequency (95% confidence interval)	^c^1/507 (1/827 - 1/311)			^c^1/2468 (1/5,522 to 1/1104)		
	^d^1/472 (1/767 - 1/291)			^d^1/2095 (1/4,441 to 1/988)		
	^e^1/472 (1/767 - 1/291)			^e^1/907 (1/1582 - 1/520)		

*P *value	^c^*P *= 0.0004; ^d^*P *= 0.0004; ^e^*P *= 0.076.					

**A: Size and phenotype (Krt18/Krt14 double positivity) of colonies**. At least 50 randomly selected colonies were examined for each group, except for colonies derived from basal stem/progenitor cells of parous mice, where all available colonies were used. Data represent the mean ± SEM of three independent experiments with a minimum of 10 mice (five virgins; five parous) per experiment. ^a^Colony size ≥ 20 cells; ^b^Colony size ≥ 100 cells. **B: Limiting dilution analysis of the repopulating capacity**. The basal stem/progenitor cell subpopulations from parous (*n *= 23) and age-matched virgin control (*n *= 21) mice were injected into the cleared fat pads of 3-week-old syngeneic female recipient mice in limiting dilution numbers. Epithelial outgrowths were scored 8 to 9 weeks after injection. Number of positive outgrowths of specified sizes is indicated per number of mammary fat pads injected (that is, X outgrowths per Y injections = X/Y). When analyzed statistically, as previously described [18], a significant decrease in the number of large outgrowths was observed for parous donors. ^c^Positive outgrowth defined as ≥ 25% of fat pad filled; ^d^positive outgrowth defined as ≥ 10% of fat pad filled; ^e^positive outgrowth defined as ≥ 3% of fat pad filled.

### Parity decreases the *in vivo *reconstitution efficiency of the basal stem/progenitor cell subpopulation

To test their proliferation potential *in vivo*, we transplanted basal stem/progenitor cells into deepithelialized mammary glands ("cleared fat pads") [12]. It already was demonstrated that basal CD49f^High ^cells have the highest mammary gland reconstitution ability of all mammary cell subpopulations in virgin mice [16,19]. Transplantation of FACS-isolated basal stem/progenitor cells into cleared fat pads demonstrated a significant decrease in the number of large outgrowths (≥ 10% and ≥ 25% of fat pad filled), indicating a decrease in the *in vivo *proliferation potential. Interestingly, when assessing also for rudimentary outgrowths (≥ 3% of fat pad filled), no significant difference in the number of outgrowths was observed between parous and virgin donors (Table 2B). Apart from the change in size, no qualitative differences were apparent between outgrowths from virgin and parous donors. In both cases, ductal as well as lobular structures were formed. Hence, early parity led to a reduction in *in vivo *mammary repopulating efficiency of FACS-isolated basal stem/progenitor cells, whereas their ability to differentiate into different mammary epithelial structures was maintained.

### Decrease in the proportion of Wnt4-secreting cells after early parity can explain decreased Wnt signaling and reduced proliferation capacity in basal mammary stem/progenitor cells

**Figure 6:**
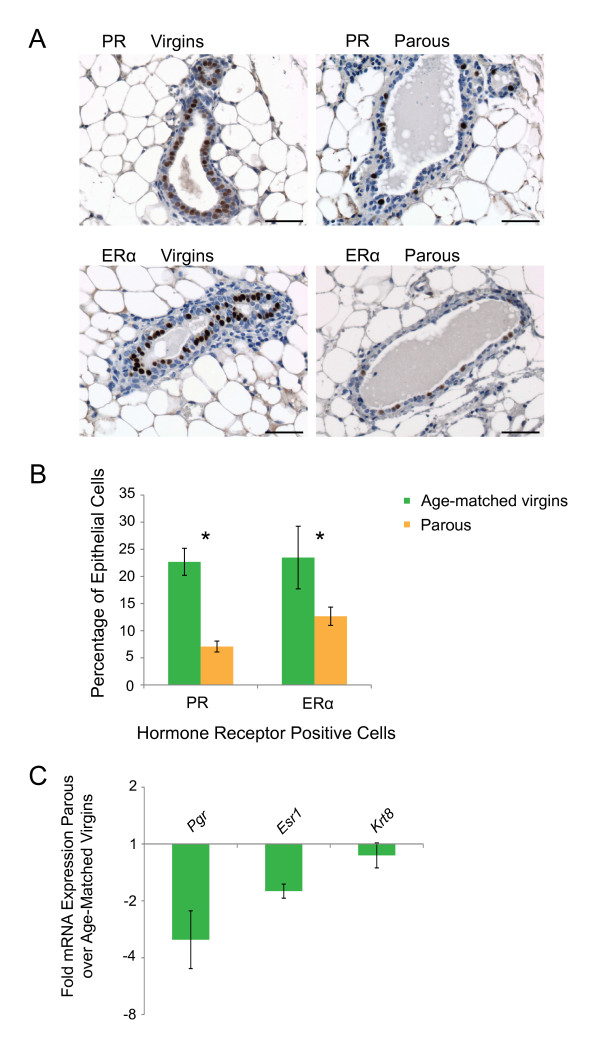
**Early pregnancy decreases the proportion of progesterone receptor (PR) and estrogen receptor α (ERα)-positive cells**. **(A) **Representative images of immunostaining for PR and ERα in mammary gland sections from age-matched virgin and parous mice in estrus. Scale bar, 50 μm. **(B) **Bar graph comparing the relative frequency of estrogen- and progesterone receptor-positive cells between mammary glands of virgin and parous mice. Data represent the mean ± SD (virgin mice, *n *= 6; parous mice, *n *= 5). For PR, *P *= 3.70E-07; for ERα, *P *= 0.003, by using two-tailed unpaired Student *t *test. **(C) **qPCR for progesterone receptor (*Pgr*), estrogen receptor alpha (*Esr1*), and the luminal marker keratin 8 (*Krt8*) genes in total mammary cell suspensions. Data are expressed as the mean ± SEM from four groups of a minimum of five parous and five age-matched virgin control mice.

**Figure 7:**
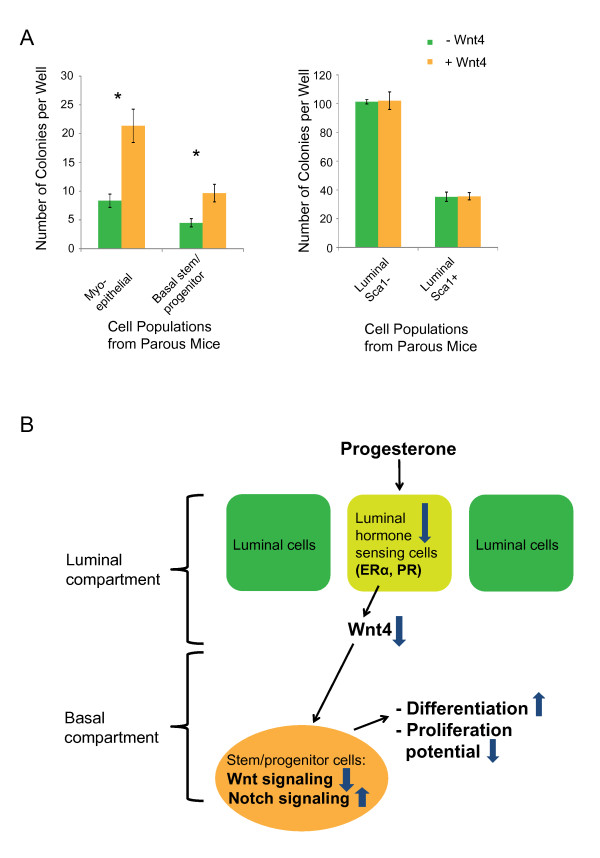
**Effect of Wnt4 on the proliferation capacity of basal stem/progenitor cells after early pregnancy in mice**. **(A) **Recombinant Wnt4 rescues the parity-induced *in vitro *proliferation defect in basal mammary epithelial cells. Selected mammary epithelial cells from parous mice were cultured in the absence or presence of recombinant Wnt4. Three independent experiments were performed. Data represent the mean ± SEM of colonies per well, with six to nine wells assessed per cell type. *P ≤ 0.02 (two-tailed unpaired Student *t *test). **(B) **Mechanistic model illustrating the parity-induced decrease in hormone-sensing and Wnt4-secreting luminal cells on the Wnt/Notch signaling pathways and the proliferation/differentiation potential in basal stem/progenitor cells.

Having observed most of the expected functional consequences of a decrease in the Wnt/Notch signaling ratio in basal stem/progenitor cells from parous mice, we finally examined the possible cause of parity-induced reduction in canonical Wnt signaling and proliferation capacity in basal stem/progenitor cells. Because parity induced a greater than threefold decrease in Wnt ligand *Wnt4 *gene expression (Figure 2A, B), and Wnt4 is known to be secreted in response to progesterone by hormone-sensing luminal cells [46], thus inducing canonical Wnt signaling in mammary stem/progenitor cells [47], a parity-induced decrease in estrogen/progesterone-sensitive luminal cells could explain the overall decrease in Wnt signaling in mammary stem cells. This hypothesis is supported by the reduction in the proportion of luminal Sca1^+ ^cells isolated from parous mice (Figure 1D) and by the demonstration that luminal Sca1^+ ^cells are hormone receptor positive (Additional file 6) [17]. Furthermore, immunohistochemical analysis of mammary gland sections for estrogen receptor alpha (ERα) and its target progesterone receptor (PR) showed a twofold decrease in ERα- and a threefold decrease in PR-positive cells in parous compared with age-matched virgin control mice (Figure 6A, B). These results were additionally verified by qPCR in total mammary cell suspensions (Figure 6C). Notably, expression of the luminal marker *Krt8 *was similar in cell suspensions from parous and age-matched virgin control mice, supporting the conclusion of a specific decrease in hormone receptor-positive cells rather than a general cell loss after pregnancy. Furthermore, parity-induced reduction in progesterone-stimulated *Wnt4 *expression was independent of blood progesterone, because average blood progesterone concentrations were similar in parous mice and age-matched virgin control mice in estrus (see Additional file 9). Finally, supplementation of the culture medium with recombinant Wnt4 stimulated *in vitro *proliferation capacity of basal myoepithelial and basal stem/progenitor cells from parous mice by +138% ± 22% and +140% ± 17%, respectively (Figure 7A). In contrast, no significant effects of recombinant Wnt4 on the colony-formation capacity of luminal Sca1^- ^(+3.7% ± 3.0%) and luminal Sca1^+ ^cells from parous mice (-5.1% ± 2.9%) were observed. These data strongly suggest a causal relation between reduced number of luminal progesterone receptor-positive/Wnt4-secreting cells and decreased Wnt/Notch signaling and proliferation potential of basal stem/progenitor cells after early pregnancy (Figure 7B).

In conclusion, all collected data indicate that early parity induces a decrease in luminal hormone-sensing ERα- and PR-positive cells, which leads to decreased *Wnt4 *expression levels and to reduced Wnt signaling in basal stem/progenitor cells. Consistent with the decrease in the Wnt/Notch signaling ratio, differentiation is promoted and proliferation inhibited in basal stem/progenitor cells of parous mice.

## Discussion

This study demonstrates that a history of early pregnancy changes the gene-expression profiles and functional properties of mammary epithelial cell subpopulations in a cell subtype-specific fashion. Most important, the following parity-induced alterations were observed in mice: (a) an induction of differentiation and downregulation of the Wnt/Notch signaling ratio in basal stem/progenitor cells; (b) a decrease in the *in vitro *and *in vivo *proliferation potential of isolated basal stem/progenitor cells; (c) a selective downregulation of potentially tumorigenic biofunctions in the basal stem/progenitor cell subpopulation; (d) a reduction in estrogen- and progesterone-responsive and Wnt4-secreting luminal cells; and (e) a rescue of the proliferation defect in basal stem/progenitor cells *in vitro *by recombinant Wnt4. The finding of a decreased Wnt/Notch signaling ratio provides direct experimental evidence for the hypothesis that early pregnancy changes the "genomic signature" of mammary stem/progenitor cells [7], causing their differentiation and reducing their proliferation potential. Furthermore, the data indicate a novel causal relation between parity-induced reduction in hormone-sensing and Wnt4-secreting luminal cells and altered biofunctions in basal stem/progenitor cells.

The basal CD49f^High ^cells, as isolated in this study, are a subfraction of basal epithelial cells [19,21]. Basal CD49f^High ^cells have been demonstrated previously to be enriched in mammary repopulating units (MRUs) (known as mammary stem cells (MaSCs)) [16,19] and to correspond to the Lin^- ^CD24^+ ^CD29^High ^epithelial cell subpopulation isolated by an alternative method [18,19,45]. However, the isolated basal CD49f^High ^epithelial cell subpopulation represents a heterogeneous cell fraction containing, in addition to MaSCs, basal progenitor cells and possibly mature myoepithelial cells [45]. Progenitor cells can be characterized *in vitro *by their colony-formation capacity [22,23], whereas MaSCs have traditionally been defined by their *in vivo *regenerative capacity [16-18]. The observed effects of parity on the *in vitro *and *in vivo *proliferation capacity of the CD49f^High ^cell subpopulation (Table 2) suggests that both basal progenitor cells and basal MaSCs are the target of an early pregnancy within the mammary epithelium. Although the dramatic decrease in the *in vitro *proliferation capacity of the CD49f^High ^cell subpopulation (Figure 5) indicates a predominant effect of an early pregnancy on basal progenitor cells, the additional reduction in large *in vivo *outgrowths (≥ 10% of fat pad filled) and the prevalence of rudimentary outgrowths (≥ 3% of fat pad filled) after parity (Table 2) suggest that isolated basal MaSCs are also affected by an early pregnancy. Because basal mammary stem and progenitor cells are closely related and likely to be interdependent in their proliferation potentials, our data do not permit a definite discrimination between basal stem and progenitor cells as primary targets of pregnancy. Therefore, we adhered to the combined term basal stem/progenitor cells throughout this study.

We found the p53-p21 pathway to be enriched to a similar extent in all mammary epithelial cell subpopulations (Additional file 7) [39], and hence, although parity-caused induction of the p53-p21 pathway may explain the relatively modest decrease in *in vitro *colony-formation potential of the luminal Sca1^+ ^cell subpopulation (Figure 5), it may contribute but cannot account for the almost complete proliferation block in basal stem/progenitor cells. The most prominent parity-induced alterations in gene expression in basal stem/progenitor cells were downregulation of the Wnt-signaling pathway, upregulation of the Notch-signaling pathway, and upregulation of differentiation genes. Decreased Wnt signaling in basal stem/progenitor cells from parous mice was verified on the protein level by measuring versican and nuclear β-catenin expression (Figures 3D and 4B, C). Wnt signaling has been shown to promote long-term expansion of cultured Lin^- ^CD24^+ ^CD29^High ^cells and to provide a competitive advantage in mammary gland reconstitution assays [43]. The latter is especially true for the expression of the classic Wnt target *Lgr5 *[48], which was found in our study to be downregulated in the basal stem/progenitor cell subpopulation after parity. Notably, Wnt signaling inhibition was demonstrated to have an antiproliferative effect in CD29^High ^cells [43]. Furthermore, Notch signaling was observed to reduce *in vitro *and *in vivo *proliferation of CD29^High ^cells while promoting their differentiation [44,49]. Because the differentiation processes of stem and progenitor cells in many organs and in several model systems are dependent on the Wnt/Notch signaling ratio [50], an overall reduction in Wnt/Notch signaling ratio would be expected to have a dramatic antiproliferation and prodifferentiation effect in mammary basal stem/progenitor cells. This is exactly what we observed in the CD49f^High ^cell subpopulation of parous mice. Thereby, the overall conclusion of our study is strengthened by the fact that all assays used (that is, transcriptome analysis, bioinformatics transcription factor, and gene-enrichment analyses, *in vitro *colony-forming assay, *in vivo *transplantation assay, and immunohistochemistry) pointed into the same direction. Thus, analysis of specific mammary epithelial cell subpopulations allowed the discovery of a decrease in the Wnt/Notch signaling ratio, which so far is the only plausible explanation for the observed differentiation burst and dramatic proliferation block experienced by basal stem/progenitor cells of parous mice.

As possible explanations for the parity-induced decrease in Wnt signaling, we found a marked increase in the activity of the Wnt repressor TCF3 in basal stem/progenitor cells and a more than threefold reduction in expression of the secreted Wnt ligand *Wnt4 *in total mammary cells from parous mice. The latter corresponded to a similar decrease in Wnt4-secreting [46] and estrogen and progesterone receptor-positive luminal cells. Notably, a similar decrease in progesterone receptor α-positive cells after parity has also been observed in human breast epithelium [51]. Hence, mechanistically, early parity decreases the hormone responsiveness of the mammary gland in mice by decreasing the number of estrogen/progesterone receptor-positive luminal cells. This reduces the paracrine signaling cascade mediated by Wnt4, inducing TCF3-dependent repression [52] and/or primary downregulation [47] of canonical Wnt signaling and secondary (reactive) upregulation of Notch signaling in basal stem/progenitor cells.

As a final consequence, proliferation is repressed and basal stem/progenitor cells differentiate. This mechanistic model is supported by the ability of recombinant Wnt4 to rescue the proliferation defect of basal stem/progenitor cells from parous mice *in vitro *(Figure 7).

Our findings in specific mammary epithelial cell subpopulations are in part consistent with and in part contradictory to studies in entire breasts/mammary glands, total mammary cells, or total mammary epithelial cells. With respect to the transcriptome analysis, our studies in total mammary cells agree with previous reports in the entire mammary glands [9,10]. However, in intact mammary glands or total mammary cells, the additional presence of stromal and dominant epithelial cell subtypes might mask the detection of key signaling-pathway changes. Indeed, our study demonstrates that isolation of specific mammary epithelial cell subpopulations is a prerequisite for the detection of a decrease in Wnt/Notch signaling ratio in basal stem/progenitor cells. A similar masking effect by stromal and dominant epithelial cell subtypes (for example, strong clonogenic luminal Sca1^- ^cells) might also explain why a previous study with a similar early-pregnancy protocol did not observe a parity-induced reduction in *in vitro *proliferation of total mixed mammary cells [20]. Controversial results have also been reported with respect to the effect of parity on the *in vivo *mammary-repopulating capacity. Hence, although Britt *et al. *[21] found no effects of late pregnancy (9 weeks) on mammary-repopulating units (MRUs) in total mammary epithelial cells, Siwko *et al. *[20] (early-pregnancy protocol) observed a parity-induced reduction in the mammary-repopulating capacity of total mixed mammary cells. With a similar early-pregnancy protocol and the same cut-off for mammary gland outgrowth (≥ 10% of fat pad filled), our findings in isolated mammary basal stem/progenitor cells appear consistent with the observations of Siwko *et al*. However, given that our studies were performed with isolated mammary basal stem/progenitor cell subpopulations, our findings are not directly comparable with and neither confirm nor contradict previous studies using total mammary (epithelial) cells [20,21]. Furthermore, the fact that the number of smaller outgrowths (≥ 3% of fat pad filled) was unchanged after parity suggests that MRUs survive after pregnancy despite their reduced reconstitution efficiency. This conclusion is in line with the recent demonstration that Wnt-responsive mammary epithelial stem cells persist after parity in *Axin2 *reporter mice [15].

It is intriguing to speculate that marked growth inhibitory effects and the downregulation of canonical Wnt signaling in basal stem/progenitor cells account, at least in part, for the cancer-protective effect of early pregnancy. Increases in canonical Wnt signaling have been linked repeatedly to oncogenesis [53,54]. Moreover, downregulation of the Wnt inhibitory protein Sfrp1 and overexpression of the Wnt target versican have been associated with carcinogenesis [55,56]. In the transcriptome analysis reported here, *Sfrp1 *was upregulated and versican downregulated, thus supporting a parity-induced anticarcinogenic effect. Moreover, Li *et al. *[57] showed that transgenes encoding components of the Wnt signaling pathway preferentially induce mammary cancers from progenitor cells. Hence, the contribution of decreased canonical Wnt signaling in basal stem/progenitor cells to the cancer-protective effect of early pregnancy may be in conjunction with other tumor-suppressing mechanisms, such as parity-induced induction of p53 [58,59]. Thereby, the increase in the TCF3 repressor activity in basal stem/progenitor cells is expected to elevate the threshold further for the activation of tumorigenic Wnt signaling [52]. In addition to decreased Wnt signaling in basal stem/progenitor cells, decreased ERα- and PR-positive cells could also be a mechanism for the breast cancer-protective effect of an early pregnancy. This is especially relevant, given the specific protective effect of pregnancy against ER/PR-positive tumors [60].

Furthermore, mammary epithelial cell differentiation *per se *has been suggested to exert a breast cancer-protective effect. This has been challenged, however, by the observation that differentiation-causing agents such as placental lactogen and perphenazine failed to protect against carcinogenesis in rodents [3,61]. Also, the hypothesis of a potential breast cancer-protective effect of mammary stromal cells [62] is not supported by our study, because stromal cells exhibited by far the fewest parity-induced gene-expression changes. However, our stromal cell subpopulation was not homogenous and devoid of immune cells, which may have masked some parity-induced alterations. In any case, the parity-induced downregulation of the Wnt/Notch signaling ratio in basal stem/progenitor cells represents a possible important mechanism for the breast cancer-protective effect of early pregnancy.

## Conclusions

This study identified downregulation of the Wnt/Notch signaling ratio in basal stem/progenitor cells as the dominant early parity-induced alteration of gene expression in mice. This change in gene expression is specific for basal mammary stem/progenitor cells, is associated with proliferation defects *in vitro *and *in vivo*, and is probably caused by an early parity-induced decrease in hormone-sensitive and Wnt4-secreting luminal cells. Importantly, because a similar reduction in progesterone receptor α-positive luminal cells has been reported in women [51], parity-induced alterations in Wnt/Notch signaling pathways may also occur in human basal stem/progenitor cells. Testing whether Wnt inhibitors mimic early parity-induced breast cancer protection warrants further investigation.

## Abbreviations

ER: estrogen receptor; FDR: false discovery rate; FWER: family-wise error rate; GSEA: gene-set enrichment analysis; MARA: motif activity response analysis; MaSC: mammary stem cell; MRU: mammary repopulating unit; NES: normalized enrichment score; PR: progesterone receptor; RBC: red blood cell; WBC: white blood cell.

## Competing interests

The authors declare that they have no competing interests.

## Authors' contributions

FM-A conceived of the hypothesis, prepared the mice, designed and performed the experiments, and wrote the manuscript. EM introduced FM-A to the techniques and assisted with some of the experiments. TR oversaw the microarray studies and assisted in the analysis of the microarray data. HB provided technical assistance with the immunohistochemistry. SD provided technical and intellectual support. DM provided technical assistance with the transplantation experiments, and IK performed fat-pad clearings and cell injections. PB and EvN performed the MARA analysis and provided intellectual support. MB-A conceived of the hypothesis, directed the project, and revised the manuscript. All authors read and approved the final manuscript.

## Supplementary Material

Additional file 1 **Flow-cytometric separation of mammary epithelial cells from virgin control and parous mice**. Representative flow-cytometry pseudocolor plots depicting the first steps in the gating strategy used to eliminate doublets, cell clumps, dead cells (DAPI bright) and white blood cells (CD45^+^) during the procedure for isolating mammary epithelial cell subpopulations from virgin control **(A) **and parous **(B) **mice. Subsequent isolation steps are shown in Figure 1. **(C) **Bar graph showing the proportion of mammary epithelial cells relative to the total white blood cell depleted (CD45^-^) mammary cells. The apparent modest decrease in total epithelial cells from parous mice was not significant (*P *= 0.05 by using two-tailed unpaired Student *t *test). Click here for file

Additional file 2 **Basal stem/progenitor cells from parous mice show reduced *in vivo *proliferation potential**. Examples of an outgrowth (left) and of no outgrowth (right) from basal stem/progenitor cells of age-matched virgin control mice and of parous mice, respectively. Click here for file

Additional file 3 **Custom gene set of Wnt target genes**. The gene set is composed of the Wnt targets that have been reported to be upregulated on canonical Wnt signaling in mammalian systems [64]. Click here for file

Additional file 4 **Verification of luminal/basal origin and purity of isolated mammary epithelial cell subpopulations. (A) **Immunofluorescent staining of isolated mammary epithelial cells with the luminal marker keratin 18 (Krt18) and the basal marker keratin 14 (Krt14). Basal myoepithelial cells were negative for Krt18 and positive for Krt14 in > 95% of total cells. Conversely, luminal Sca1^- ^and luminal Sca1^+ ^cells were positive for Krt18 and negative for Krt14 in > 95% of total cells. These data confirm the basal and luminal origin of the isolated cell subpopulations. Basal stem/progenitor cells were positive for Krt14 and Krt18 in > 95% and about 20% of total cells, respectively. Data are representative of three independent experiments. Scale bar, 50 μm. (**B/C) **qPCR for *CD49f *and *Sca1 *in FACS-sorted mammary epithelial cell subpopulations. Fold changes are shown relative to myoepithelial cells. Data are expressed as the mean ± SEM of three independent experiments. Click here for file

Additional file 5 **Control for complete involution**. Representative images of whole mounts of mammary glands from virgins and parous mice 28 and 40 days after weaning. Mammary glands were completely involuted at 28 days and certainly at 40 days after weaning. Click here for file

Additional file 6 **Influence of cell number on transcriptome analysis and validation of the amplification method. (A) **Pairwise correlation plot of transcriptome data derived from 2,000 and 50,000 myoepithelial and luminal Sca1^- ^cells, and from 2,000 basal CD49f^High ^stem/progenitor cells isolated from 11-week-old virgin mice (*n *= 6). Individual arrays were pairwise correlated by using the unfiltered data as input. Pearson correlation coefficients were calculated and mapped onto a gray scale from black (low values) to white (high values). Higher cell numbers resulted in higher reproducibility, as assessed by high Pearson correlation coefficients. However, although lower cell numbers resulted in lower Pearson correlation coefficients, all arrays of one cell subpopulation were clearly discernible from other subpopulations, irrespective of the cell number used. Thus, in the range of 2,000 to 50,000 cells, cell-subpopulation identity was more determining for cluster analyses than cell number. **(B) **qPCR on amplified cDNA of mammary epithelial cell subpopulations. Data were normalized to the reference gene *B2M *and are shown relative to 50,000 cells of myoepithelial cells. The basal marker keratin 14 (*Krt14*) was expressed by myoepithelial cells and basal CD49f^High ^stem/progenitor cells, but not by luminal Sca1^- ^and luminal Sca1^+ ^cells. Conversely, the luminal markers keratin 8 (*Krt8*) and keratin 19 (*Krt19*) were expressed by luminal Sca1^- ^and luminal Sca1^+ ^cells, but not by myoepithelial and not by basal CD49f^High ^stem/progenitor cells. As expected, the estrogen receptor alpha (*Esr1*) was expressed by luminal Sca1^+ ^cells only. Data represent the means of duplicates. **(C) **qPCR on unamplified cDNA. Data were processed and analyzed as in **(B)**. Changes in expression levels of the luminal and basal markers *Krt19 *and *Krt14*, respectively, were similar to those of amplified cDNA, indicating that the amplification process was unbiased. Click here for file

Additional file 7 **The decrease in Wnt signaling is specific for basal stem/progenitor cells, whereas the p53-p21 pathway is upregulated to the same degree in all mammary epithelial cell subpopulations from parous mice. (A) **Enrichment plots of Wnt target gene-set enrichment analysis [28,29] for isolated mammary epithelial cell subpopulations. The enrichment score is plotted against the ranked gene list, calculated by subtracting the gene expression levels of cells from age-matched virgins and parous mice. The gene set contained all canonical Wnt target genes reported in mammalian systems (Additional file 3). Thus, positive enrichment scores indicate an upregulation, and negative enrichment scores, a downregulation of Wnt signaling in cells of parous mice. Statistical analysis indicated specific downregulation of Wnt signaling in basal stem/progenitor cells from parous mice (Table 1). The apparent downregulation of Wnt signaling in myoepithelial cells, which are contaminated with basal stem/progenitor cells, was not significant. **(B) **Bar plot of gene-set enrichment analysis (GSEA)-calculated and normalized enrichment scores for the p53-p21 pathway previously identified by Sivaraman *et al. *[39]. The p53-p21 gene set was rendered by GSEA as significantly upregulated in all isolated mammary epithelial cell subpopulations from parous mice when testing for all signaling-pathway gene sets contained in v2.5 and v3.0. The nominal *P *value, the false discovery rate (FDR), and the family-wise error rate (FWER) for this pathway were < 0.01 for all mammary epithelial cell subpopulations; 1,000 permutations were performed with the permutation type "gene set." **(C) **Representative images of immunostaining for p21 and bar graph comparing the relative frequency of p21-positive epithelial cells in mammary gland sections from age-matched virgin and parous mice in estrus. Data represent the mean ± SD (virgin mice: *n *= 3; parous mice: *n *= 3). *P *= 0.0007, by using two-tailed unpaired Student *t *test. Scale bar, 25 μm. Click here for file

Additional file 8 **Twenty most significantly downregulated pathways in basal stem/progenitor cells after parity**. The list was calculated by using v3.0 of GSEA [28]. The 1,000 permutations were performed by using the permutation type "gene set." In all other cases, the default settings were used. Click here for file

Additional file 9 **Blood progesterone concentrations in parous and age-matched virgin control mice in estrus do not change significantly**. Plasma progesterone levels were measured with ELISA. Click here for file
