# Supplementary figures and images for: Parity induces differentiation and reduces Wnt/Notch signaling ratio and proliferation potential of basal stem/progenitor cells isolated from mouse mammary epithelium
Source: Breast Cancer Res. 2013 Apr 29;15(2):R36. doi: 10.1186/bcr3419 (PMC3672662; doi:10.1186/bcr3419)

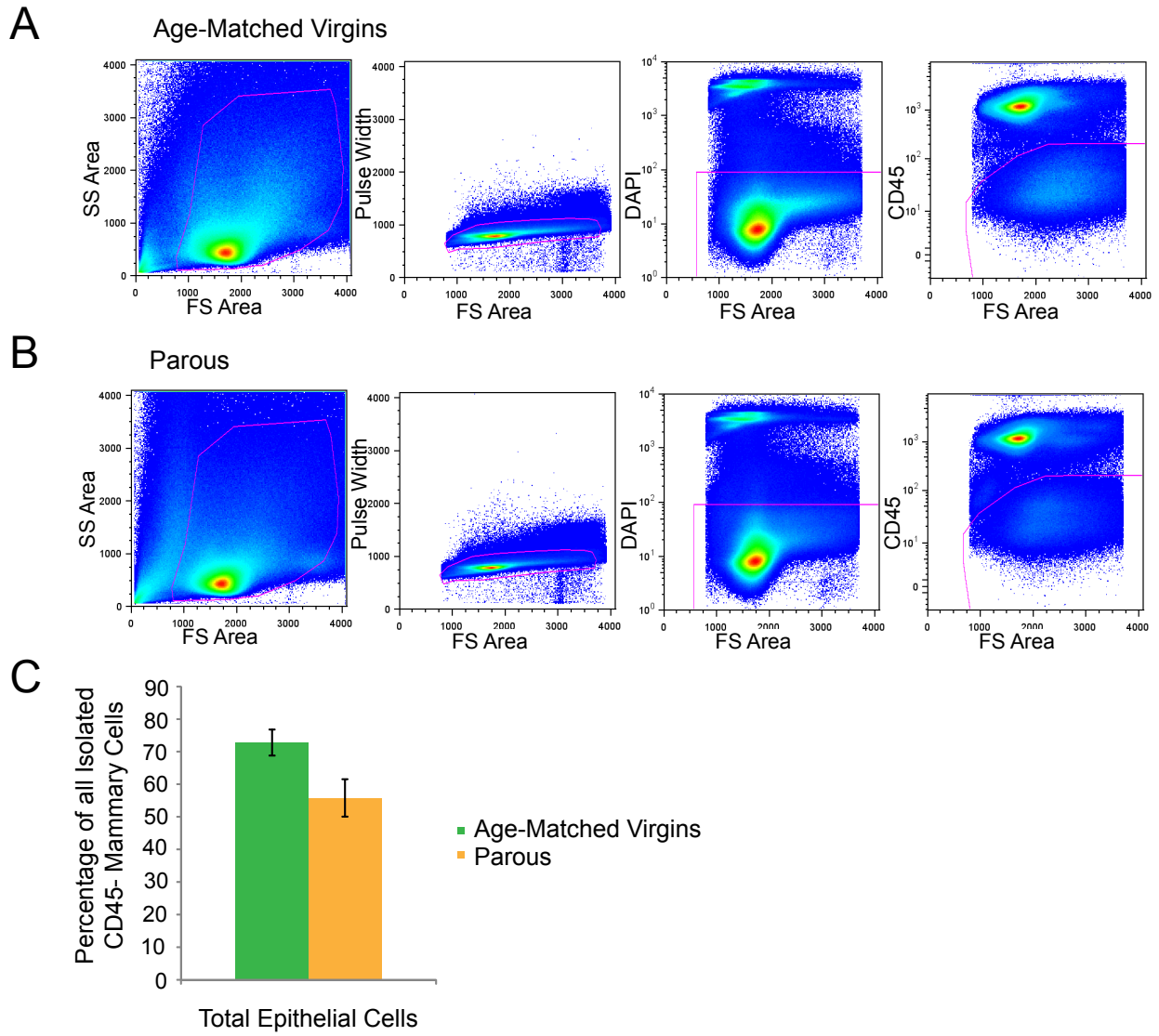

Supplement: Additional file 1 — Flow-cytometric separation of mammary epithelial cells from virgin control and parous mice. Representative flow-cytometry pseudocolor plots depicting the first steps in the gating strategy used to eliminate doublets, cell clumps, dead cells (DAPI bright) and white blood cells (CD45+) during the procedure for isolating mammary epithelial cell subpopulations from virgin control (A) and parous (B) mice. Subsequent isolation steps are shown in Figure 1. (C) Bar graph showing the proportion of mammary epithelial cells relative to the total white blood cell depleted (CD45-) mammary cells. The apparent modest decrease in total epithelial cells from parous mice was not significant (P = 0.05 by using two-tailed unpaired Student t test). [file bcr3419-S1.PDF]

## Additional file 2

Meier-Abt et al.

Outgrowth from virgin donors

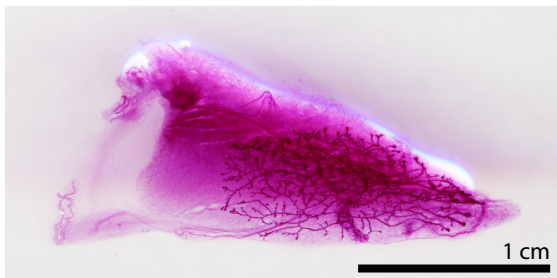

No outgrowth from parous donors

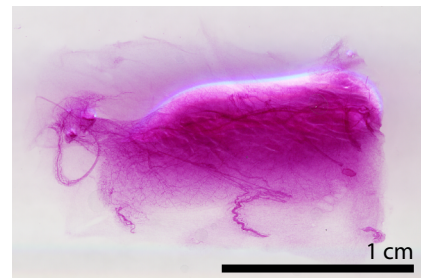

Supplement: Additional file 2 — Basal stem/progenitor cells from parous mice show reduced in vivo proliferation potential. Examples of an outgrowth (left) and of no outgrowth (right) from basal stem/progenitor cells of age-matched virgin control mice and of parous mice, respectively. [file bcr3419-S2.PDF]

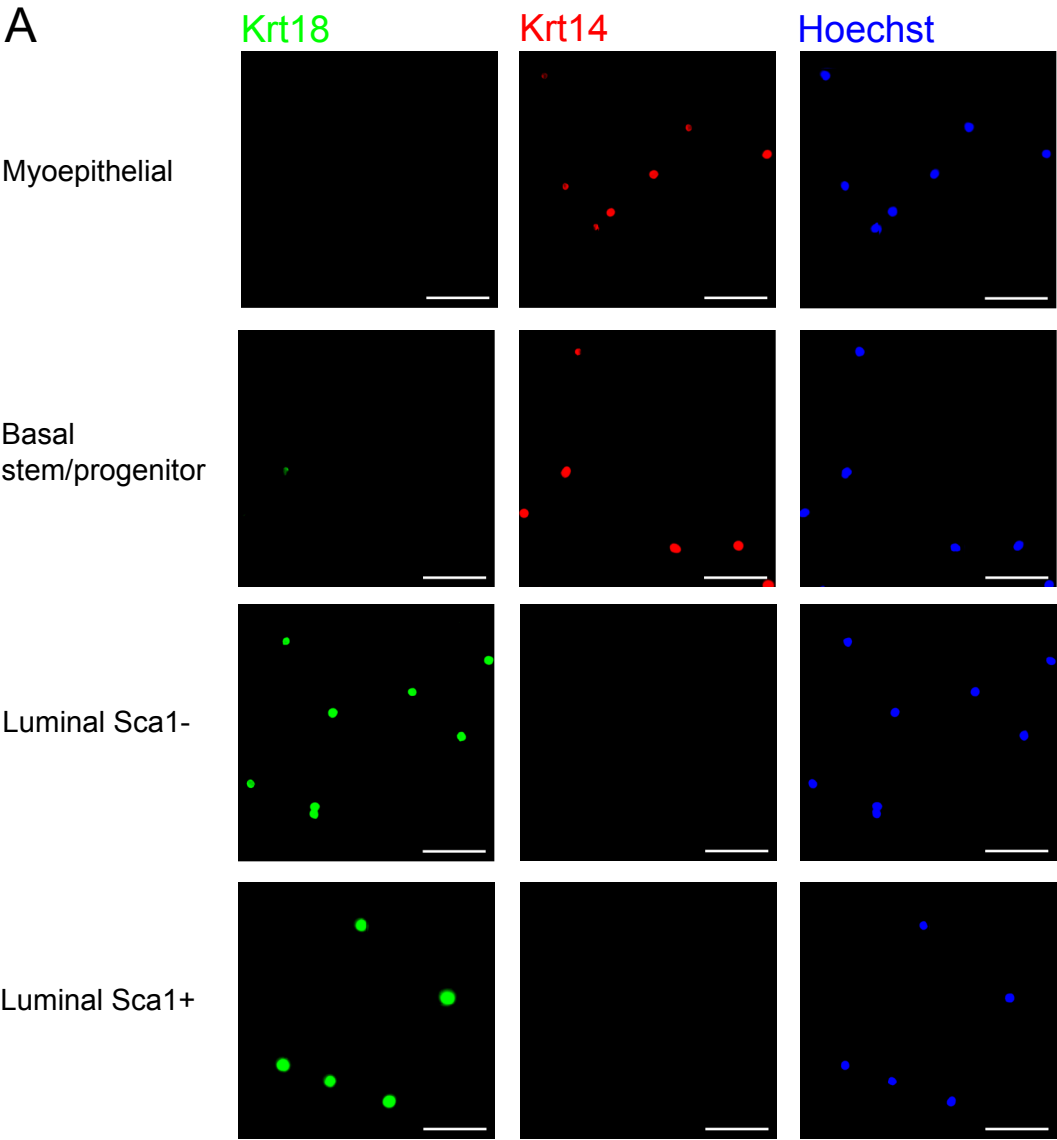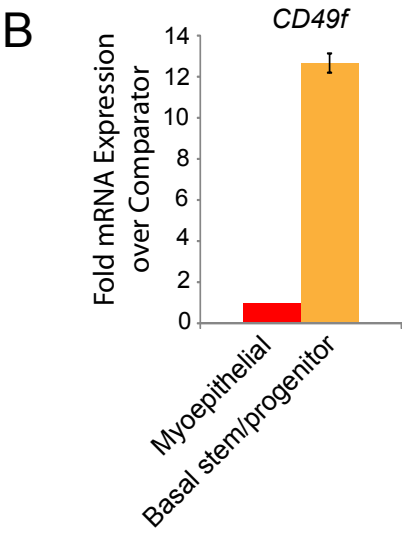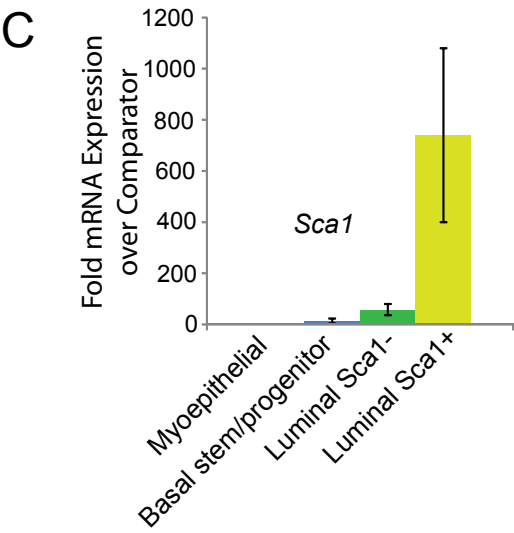

Supplement: Additional file 4 — Verification of luminal/basal origin and purity of isolated mammary epithelial cell subpopulations. (A) Immunofluorescent staining of isolated mammary epithelial cells with the luminal marker keratin 18 (Krt18) and the basal marker keratin 14 (Krt14). Basal myoepithelial cells were negative for Krt18 and positive for Krt14 in > 95% of total cells. Conversely, luminal Sca1- and luminal Sca1+ cells were positive for Krt18 and negative for Krt14 in > 95% of total cells. These data confirm the basal and luminal origin of the isolated cell subpopulations. Basal stem/progenitor cells were positive for Krt14 and Krt18 in > 95% and about 20% of total cells, respectively. Data are representative of three independent experiments. Scale bar, 50 μm. (B/C) qPCR for CD49f and Sca1 in FACS-sorted mammary epithelial cell subpopulations. Fold changes are shown relative to myoepithelial cells. Data are expressed as the mean ± SEM of three independent experiments. [file bcr3419-S4.PDF]

## Additional file 5

Meier-Abt et al.

Virgin

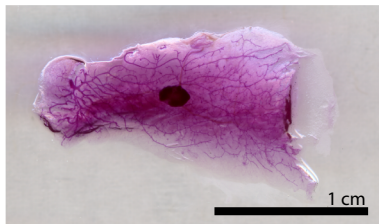

28 days post weaning

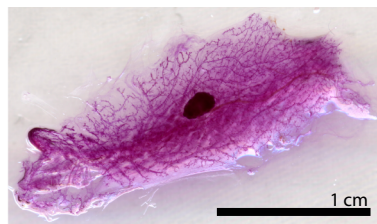

40 days post weaning

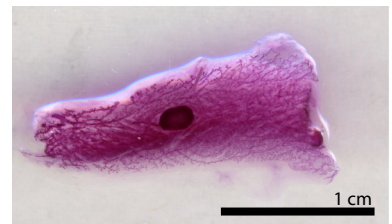

Supplement: Additional file 5 — Control for complete involution. Representative images of whole mounts of mammary glands from virgins and parous mice 28 and 40 days after weaning. Mammary glands were completely involuted at 28 days and certainly at 40 days after weaning. [file bcr3419-S5.PDF]

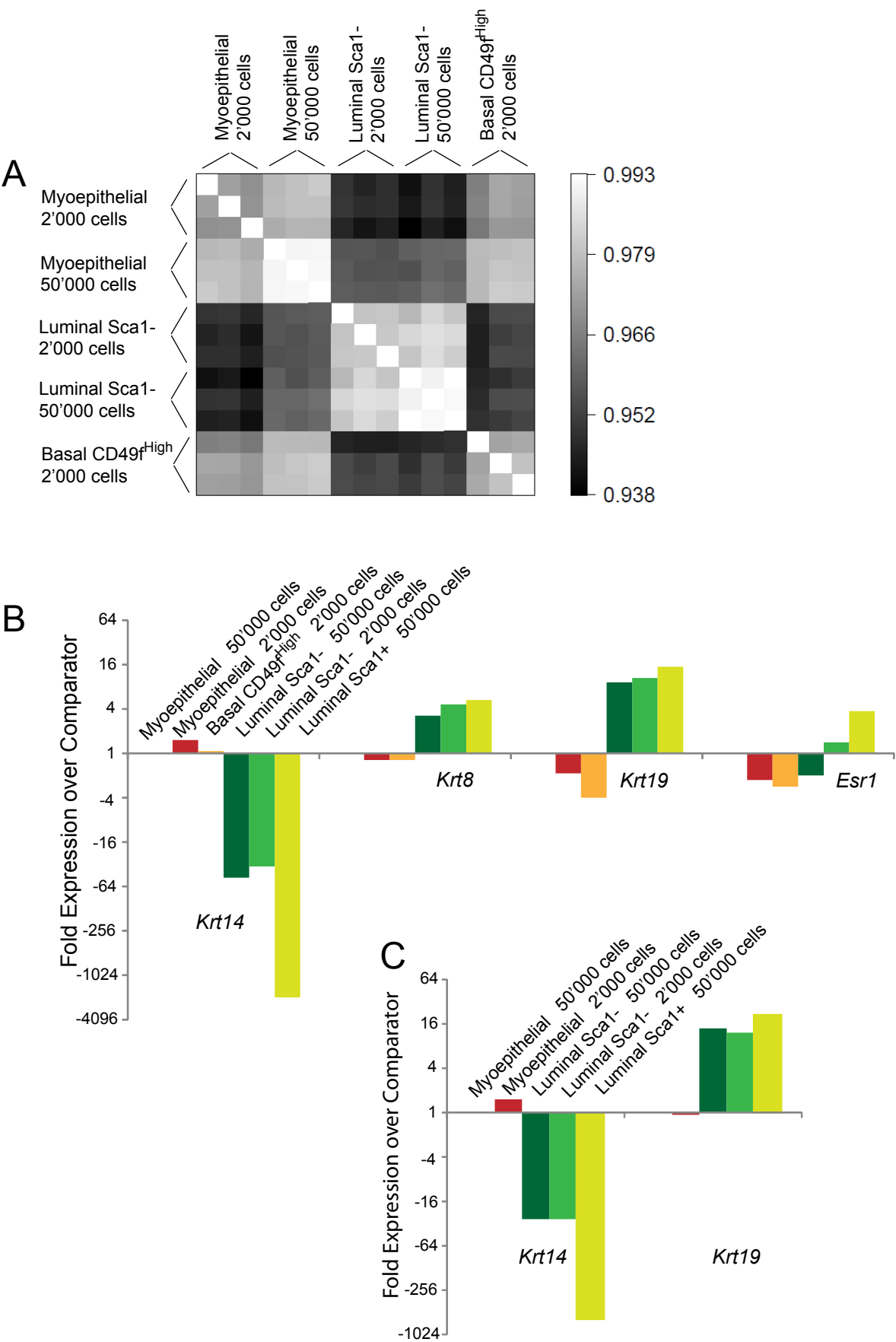

Supplement: Additional file 6 — Influence of cell number on transcriptome analysis and validation of the amplification method. (A) Pairwise correlation plot of transcriptome data derived from 2,000 and 50,000 myoepithelial and luminal Sca1- cells, and from 2,000 basal CD49fHigh stem/progenitor cells isolated from 11-week-old virgin mice (n = 6). Individual arrays were pairwise correlated by using the unfiltered data as input. Pearson correlation coefficients were calculated and mapped onto a gray scale from black (low values) to white (high values). Higher cell numbers resulted in higher reproducibility, as assessed by high Pearson correlation coefficients. However, although lower cell numbers resulted in lower Pearson correlation coefficients, all arrays of one cell subpopulation were clearly discernible from other subpopulations, irrespective of the cell number used. Thus, in the range of 2,000 to 50,000 cells, cell-subpopulation identity was more determining for cluster analyses than cell number. (B) qPCR on amplified cDNA of mammary epithelial cell subpopulations. Data were normalized to the reference gene B2M and are shown relative to 50,000 cells of myoepithelial cells. The basal marker keratin 14 (Krt14) was expressed by myoepithelial cells and basal CD49fHigh stem/progenitor cells, but not by luminal Sca1- and luminal Sca1+ cells. Conversely, the luminal markers keratin 8 (Krt8) and keratin 19 (Krt19) were expressed by luminal Sca1- and luminal Sca1+ cells, but not by myoepithelial and not by basal CD49fHigh stem/progenitor cells. As expected, the estrogen receptor alpha (Esr1) was expressed by luminal Sca1+ cells only. Data represent the means of duplicates. (C) qPCR on unamplified cDNA. Data were processed and analyzed as in (B). Changes in expression levels of the luminal and basal markers Krt19 and Krt14, respectively, were similar to those of amplified cDNA, indicating that the amplification process was unbiased. [file bcr3419-S6.PDF]

**A**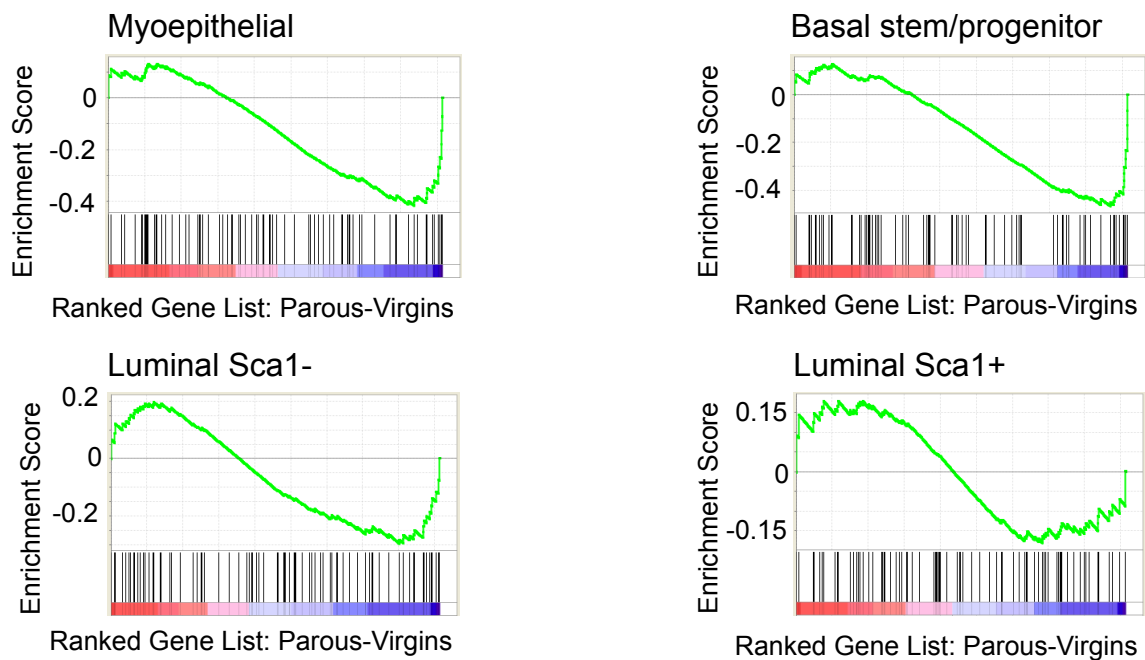**B**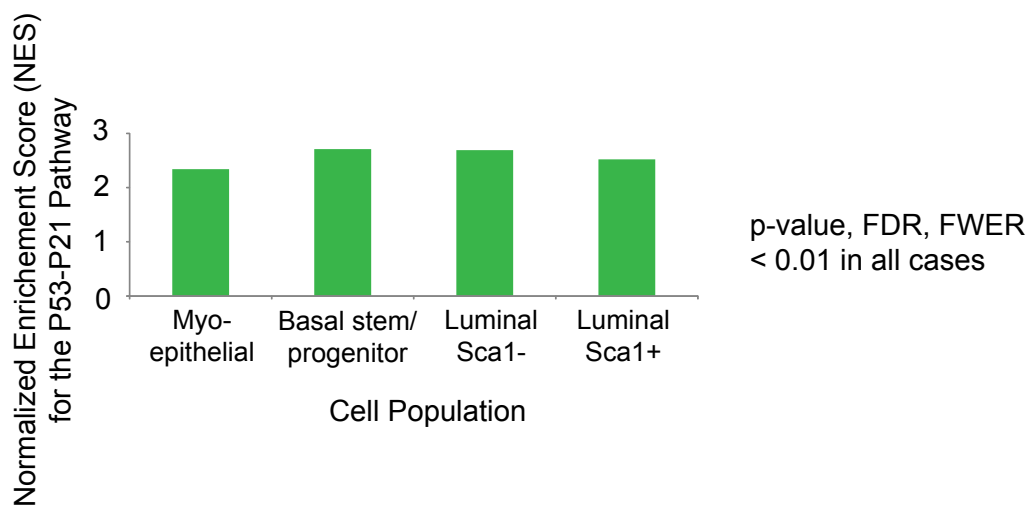**C**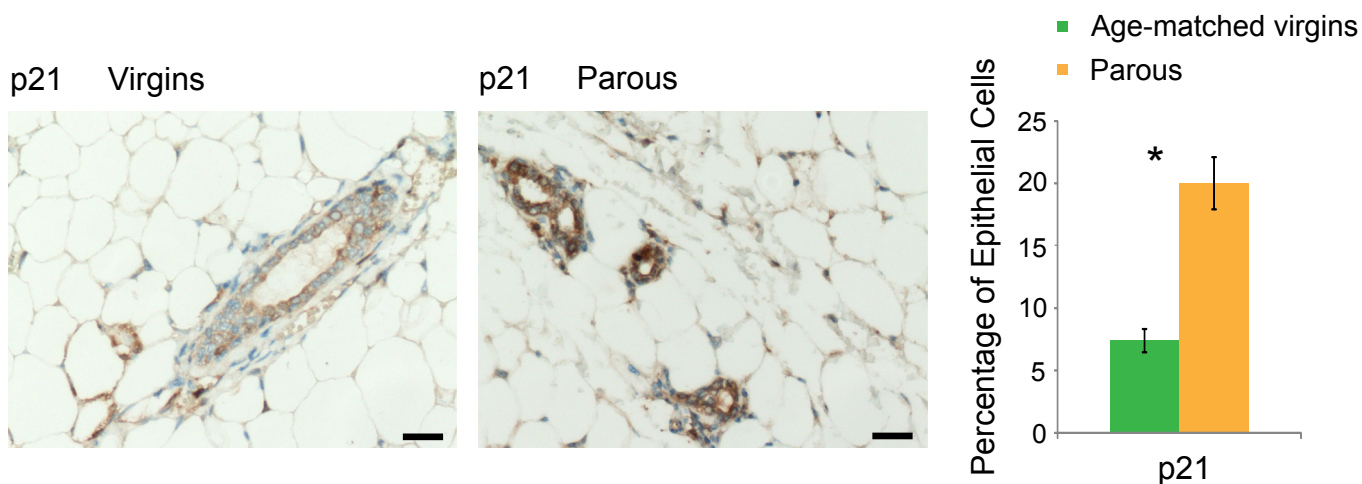

Supplement: Additional file 7 — The decrease in Wnt signaling is specific for basal stem/progenitor cells, whereas the p53-p21 pathway is upregulated to the same degree in all mammary epithelial cell subpopulations from parous mice. (A) Enrichment plots of Wnt target gene-set enrichment analysis [28,29] for isolated mammary epithelial cell subpopulations. The enrichment score is plotted against the ranked gene list, calculated by subtracting the gene expression levels of cells from age-matched virgins and parous mice. The gene set contained all canonical Wnt target genes reported in mammalian systems (Additional file 3). Thus, positive enrichment scores indicate an upregulation, and negative enrichment scores, a downregulation of Wnt signaling in cells of parous mice. Statistical analysis indicated specific downregulation of Wnt signaling in basal stem/progenitor cells from parous mice (Table 1). The apparent downregulation of Wnt signaling in myoepithelial cells, which are contaminated with basal stem/progenitor cells, was not significant. (B) Bar plot of gene-set enrichment analysis (GSEA)-calculated and normalized enrichment scores for the p53-p21 pathway previously identified by Sivaraman et al. [39]. The p53-p21 gene set was rendered by GSEA as significantly upregulated in all isolated mammary epithelial cell subpopulations from parous mice when testing for all signaling-pathway gene sets contained in v2.5 and v3.0. The nominal P value, the false discovery rate (FDR), and the family-wise error rate (FWER) for this pathway were < 0.01 for all mammary epithelial cell subpopulations; 1,000 permutations were performed with the permutation type "gene set." (C) Representative images of immunostaining for p21 and bar graph comparing the relative frequency of p21-positive epithelial cells in mammary gland sections from age-matched virgin and parous mice in estrus. Data represent the mean ± SD (virgin mice: n = 3; parous mice: n = 3). P = 0.0007, by using two-tailed unpaired Student t test. Scale bar, [file bcr3419-S7.PDF]
