# Supplementary material for: Parity induces differentiation and reduces Wnt/Notch signaling ratio and proliferation potential of basal stem/progenitor cells isolated from mouse mammary epithelium
Source: Breast Cancer Res. 2013 Apr 29;15(2):R36. doi: 10.1186/bcr3419 (PMC3672662; doi:10.1186/bcr3419)
Supplement: Additional file 3 — Custom gene set of Wnt target genes. The gene set is composed of the Wnt targets that have been reported to be upregulated on canonical Wnt signaling in mammalian systems [64]. [file bcr3419-S3.PDF]

Additional file 3

Custom gene set of Wnt target genes

MYC
CCND1
TCF7
LEF1
PPARD
JUN
FOSL1
PLAUR
MMP7
AXIN2
NRCAM
TCF4
GAST
CD44
CLDN1
BIRC5
VEGFA
FGF18
ATOH1
MET
EDN1
MYCBP
L1CAM
ID2
JAG1
TIAM1
NOS2
DKK1
FGF9
LBH
FGF20
LGR5
SOX17
RUNX2
GREM1
SALL4
CYR61
SOX2
PTTG
DLL1
FOXN1
MMP26
NANOG
POU5F1
SNAI1/2
FN1
FZD7
FST
WNT3A
ISL1
MMP2
MMP9
EN2
STRA6
EFNB1
ENPP2
ISLR
TWIST1
MMP3
T
GCG
CDX1
PTGS2
IRX3
SIX3
NEUROG1
SP5
NEUROD1
GBX2
WISP1
WISP2
IGF2
IGF1
VEGFC
IL6
CDX1
CDX4
SFRP2
PITX2
EGFR
EDA
KRT15
OVOL1
CTLA4
FGF4
IL8
RET
GJA1
VCAN
TNFRSF19
IGFBP3
