# Supplementary material for: Parity induces differentiation and reduces Wnt/Notch signaling ratio and proliferation potential of basal stem/progenitor cells isolated from mouse mammary epithelium
Source: Breast Cancer Res. 2013 Apr 29;15(2):R36. doi: 10.1186/bcr3419 (PMC3672662; doi:10.1186/bcr3419)
Supplement: Additional file 8 — Twenty most significantly downregulated pathways in basal stem/progenitor cells after parity. The list was calculated by using v3.0 of GSEA [28]. The 1,000 permutations were performed by using the permutation type "gene set." In all other cases, the default settings were used. [file bcr3419-S8.PDF]

Twenty most significantly downregulated pathways in basal stem/progenitor cells after parity

Gene set name	NES	NOM p-value	FDR q-value	FWER p-value
KOBAYASHI_EGFR_SIGNALING_24HR_DN	-3.10156	<0.01	<0.01	<0.01
SOTIRIOU_BREAST_CANCER_GRADE_1_VS_3_UP	-3.09002	<0.01	<0.01	<0.01
ROSTY_CERVICAL_CANCER_PROLIFERATION_CLUSTER	-3.03387	<0.01	<0.01	<0.01
SHEDDEN_LUNG_CANCER_POOR_SURVIVAL_A6	-2.91205	<0.01	<0.01	<0.01
HOFFMANN_LARGE_TO_SMALL_PRE_BII_LYMPHOCYTE_UP	-2.87939	<0.01	<0.01	<0.01
GRAHAM_CML_DIVIDING_VS_NORMAL_QUIESCENT_UP	-2.81022	<0.01	<0.01	<0.01
FURUKAWA_DUSP6_TARGETS_PCI35_DN	-2.7968	<0.01	<0.01	<0.01
ODONNELL_TFRC_TARGETS_DN	-2.77325	<0.01	<0.01	<0.01
WINNEPENNINCKX_MELANOMA_METASTASIS_UP	-2.7715	<0.01	<0.01	<0.01
CROONQUIST_IL6_DEPRIVATION_DN	-2.76799	<0.01	<0.01	<0.01
BENPORATH_PROLIFERATION	-2.71965	<0.01	<0.01	<0.01
WHITEFORD_PEDIATRIC_CANCER_MARKERS	-2.71767	<0.01	<0.01	<0.01
CHIANG_LIVER_CANCER_SUBCLASS_PROLIFERATION_UP	-2.71177	<0.01	<0.01	<0.01
LEE_EARLY_T_LYMPHOCYTE_UP	-2.70924	<0.01	<0.01	<0.01
GRAHAM_NORMAL_QUIESCENT_VS_NORMAL_DIVIDING_DN	-2.69762	<0.01	<0.01	<0.01
KANG_DOXORUBICIN_RESISTANCE_UP	-2.6926	<0.01	<0.01	<0.01
FERREIRA_EWINGS_SARCOMA_UNSTABLE_VS_STABLE_UP	-2.6593	<0.01	<0.01	<0.01
BERENJENO_TRANSFORMED_BY_RHOA_UP	-2.64615	<0.01	<0.01	<0.01
PUJANA_BRCA2_PCC_NETWORK	-2.63882	<0.01	<0.01	<0.01
WILCOX_PRESPONSE_TO_PROGESTERONE_UP	-2.63688	<0.01	<0.01	<0.01
