# Supplementary material for: Parity induces differentiation and reduces Wnt/Notch signaling ratio and proliferation potential of basal stem/progenitor cells isolated from mouse mammary epithelium
Source: Breast Cancer Res. 2013 Apr 29;15(2):R36. doi: 10.1186/bcr3419 (PMC3672662; doi:10.1186/bcr3419)
Supplement: Additional file 9 — Blood progesterone concentrations in parous and age-matched virgin control mice in estrus do not change significantly. Plasma progesterone levels were measured with ELISA. [file bcr3419-S9.PDF]

Plasma progesterone levels in mice

	Average plasma progesterone concentration (ng/mL) ± s.d.
Age-matched virgin control mice in estrus (n=5)	**4.6 ± 1.8**
Parous mice in estrus (n=6)	**3.2 ± 0.9**
P value	**0.14**
